# Transcriptome Analysis of Salt Stress Responsiveness in the Seedlings of Dongxiang Wild Rice (*Oryza rufipogon* Griff.)

**DOI:** 10.1371/journal.pone.0146242

**Published:** 2016-01-11

**Authors:** Yi Zhou, Ping Yang, Fenglei Cui, Fantao Zhang, Xiangdong Luo, Jiankun Xie

**Affiliations:** 1 College of Life Sciences, Jiangxi Normal University, Nanchang, China; 2 Institute for Advanced Study, Jiangxi Normal University, Nanchang, China; Jawaharlal Nehru University, INDIA

## Abstract

Dongxiang wild rice (*Oryza rufipogon* Griff.) is the progenitor of cultivated rice (*Oryza sativa* L.), and is well known for its superior level of tolerance against cold, drought and diseases. To date, however, little is known about the salt-tolerant character of Dongxiang wild rice. To elucidate the molecular genetic mechanisms of salt-stress tolerance in Dongxiang wild rice, the Illumina HiSeq 2000 platform was used to analyze the transcriptome profiles of the leaves and roots at the seedling stage under salt stress compared with those under normal conditions. The analysis results for the sequencing data showed that 6,867 transcripts were differentially expressed in the leaves (2,216 up-regulated and 4,651 down-regulated) and 4,988 transcripts in the roots (3,105 up-regulated and 1,883 down-regulated). Among these differentially expressed genes, the detection of many transcription factor genes demonstrated that multiple regulatory pathways were involved in salt stress tolerance. In addition, the differentially expressed genes were compared with the previous RNA-Seq analysis of salt-stress responses in cultivated rice Nipponbare, indicating the possible specific molecular mechanisms of salt-stress responses for Dongxiang wild rice. A large number of the salt-inducible genes identified in this study were co-localized onto fine-mapped salt-tolerance-related quantitative trait loci, providing candidates for gene cloning and elucidation of molecular mechanisms responsible for salt-stress tolerance in rice.

## Introduction

Salt stress is a vital problem for plant growth and agricultural productivity. Rice (*Oryza sativa* L.) is one of the most important food crops in the world and also a model for genomic research in monocots [1]. However, salinity is one of the most devastating abiotic stresses in rice, and the salt-affected soils currently account for about 20% of the total paddy rice planting area. More seriously, the area of salt-affected irrigated land is expanding and spreading in China [2]. In recent years, many studies have provided valuable insight into the molecular and cellular mechanisms by which rice responds to and tolerate salinity [3–7]. However, the regulatory mechanisms involved in coordinating salt stress tolerance and plant growth are not fully understood.

Dongxiang wild rice (*Oryza rufipogon* Griff., hereafter referred as DXWR) is the progenitor of cultivated rice (*Oryza sativa* L.). DXWR, a Chinese type of wild rice grown in Jiangxi Province (28°14’N latitude and 116°30’E longtitude), is considered to be the northernmost region in the world where *O*. *rufipogon* is found [8]. DXWR grows in the natural habitats and possesses various characteristics resistant to biotic and abiotic stresses and abundant genetic diversity which have been lost in the cultivated rice. Thus, it is an extremely important resource for providing a valuable gene pool for rice genetic improvement. To date, numerous quantitative trait loci (QTLs) have been mined from the DXWR, which are involved in biotic and abiotic stress tolerances [9–14]. But, the instances of rice improvement in stress tolerances with the help of DXWR were still rather rare. So far, only two genes (OrbHLH001 and OrbHLH2) of DXWR were reported to be involved in the salt stress resistance [15, 16], but the molecular mechanism on the salt stress resistance of DXWR remained unclear.

Gene expression profiling is accelerating our progress toward a comprehensive understanding of the genetic mechanisms that control responses to environmental stress. Microarray analysis and tag-based sequencing approaches have been used to investigate over-all gene expression profiles in various plants [17–21]. However, these technologies have critical limitations, such as low throughput and low sensitivity [22]. Recently, next-generation high-throughput RNA sequencing technology (RNA-Seq) could overcome the drawbacks of array-based technologies. With the high resolution and sensitivity, the RNA-Seq could be used for discovering novel splice junctions, novel transcripts, alternative transcription start sites and rare transcripts [23]. Moreover, RNA-Seq data revealed a high level of reproducibility in both technical and biological replicates [24]. So far, the global gene expression in various plants has been profiled by RNA-Seq [25–29]. In addition, RNA-Seq has been applied to the identification of stress-inducible transcripts in rice [30, 31]. However, little effort is being expended in attempts to investigate stress resistances of DXWR using RNA-Seq.

Transcription factors are integral in linking salt sensory pathways to many tolerance responses. Core sets of transcription factor family genes are differentially expressed in response to elevated external salinity [32], including basic leucine zipper (bZIP) [33], WRKY [34, 35], APETALA2/ETHYLENE RESPONSE FACTOR (AP2/ERF) [36], MYB [37], basic helix-loop-helix (bHLH) [38], and NAC [39] families. These transcription factors, in turn, regulate the expression levels of various genes that may ultimately influence the level of salt tolerance of plants [40]. In rice, recent studies have indicated that a large number of transcription factors were involved in salt stress response, such as *OsMYB91* [7], *OsbZIP71* [41], *OsWRKY42* [42], *SERF1* [3], *OsTZF1* [43], and *OsNAC5* [44].

In rice, important traits such as salt-tolerance, yield and quality are controlled by polygenes or gene complexes that are described as quantitative trait loci (QTL). The recent development of rice molecular maps has facilitated the analysis of QTLs for many factors, such as yield [45], heading date [46], and seed dormancy [47]. The dissection of such a complex trait by means of the QTL mapping-approach will be of great significance for breeding by enhancing the salt tolerance of rice. QTL analyses of salt tolerance have been conducted by several researches [48, 49]. But, the identification of genes underlying QTLs could be time consuming and expensive [50]. To date, few key salt responsive genes were identified by QTLs.

In this study, we used the Illumina sequencing to perform deep transcriptome sequencing to compare the genome-wide differential expression between salt-treatment and normal condition DXWR at the seedling stage. Through identifying potential candidate genes involved in the salt stress response in DXWR, this work may provide useful information and potential genetic resources for the improvement of salt-tolerant characters in rice. Furthermore, this study may provide a starting point for the elucidation of the molecular mechanism underlying the salt stress response in DXWR.

## Materials and Methods

### Plant material and salt stress treatment

Seeds of Dongxiang wild rice (*Oryza rufipogon* Griff.; Dongxiang County, Jiangxi Province) and rice Xieqingzao B (*O*. *sativa* L. ssp. *indica*) were immersed in distilled water in the dark, and the uniformly germinated seeds were sown in 96-well plates supported by a plastic container. Seeds were grown in a growth chamber, as previously described [12]. The growth culture solution was renewed every 3 days. After the seedlings had been grown for 14 days, they were transferred on their 96-well plates into containers filled with 200 mM NaCl solution, or with control solution for 12 days. The seedlings were then recovered under normal solution for 3 days, and survival rates were calculated. The experiment was a randomized complete block design with three replications. For RNA-Seq analysis, 14-day-old seedlings of Dongxiang wild rice were grown with or without 200 mM NaCl treatment for 3 days and then the leaves (penultimate leaves) and total roots (separated from the culture solution and washed carefully) of these seedlings were collected and immediately frozen in liquid nitrogen, respectively. For RNA extraction from each treatment group, 10 plants were collected and mixed, to minimize the effect of transcriptome unevenness among plants.

### RNA extraction, cDNA library preparation and sequencing

Total RNA was extracted for the three biological replicates from the sampled leaf or root tissues collected from the ten seedlings for each biological replicate using the TRIzol kit following the manufacturer’s instructions (Invitrogen). Total RNA was then purified and concentrated using the RNeasy MinElute cleanup kit (Qiagen). The RNA quality was checked using Bioanalyzer 2100 (Agilent). Equal quantities of total RNA from three biological replicates for each tissue sample were then pooled for the following RNA sequencing. Magnetic beads with Oligo (dT) were used to isolate mRNA from the total RNA. Mixed with the fragmentation buffer, the mRNA is fragmented into short fragments. Then cDNA is synthesized using the mRNA fragments as templates. Short fragments were purified and resolved with EB buffer for end reparation and poly (A) addition. After that, the short fragments were connected with adapters. After agarose gel electrophoresis, the suitable fragments (200 bp) were selected for the PCR amplification as templates. The library was sequenced using the Illumina HiSeq^−^ 2000 platform.

### Reads filtration and assessment of differential gene expression

Before assembly, adaptor sequences, empty reads, low quality sequences with ‘N’ percentage over 10% and those containing more than 50% bases with a Q-value < 20 were removed using the Perl program written according to the custom method of Program editing. After filtering, the remaining reads were called clean reads and used for downstream bioinformatics analysis. The retained high-quality reads were mapped to the Nipponbare reference genome [51] by Tophat (-N 2-read-gap-length 3-read-edit-dist 3-read-realign-edit-dist 0-report-secondary-alignments-coverage-search-microexon-search-library-type fr-unstranded-b2-sensitive) [52], and then the resulting aligned reads were used to create a RABT (Reference Annotation Based Transcript) assembly using Cufflinks [53]. The sequence reads were submitted to GenBank GEO database under accession number GSE73181 (http://www.ncbi.nlm.nih.gov/geo/query/acc.cgi?acc=GSE73181).

Expression levels for each gene were calculated by quantifying the reads according to the RPKM (reads per kilobase per million reads) method [54]. We used ‘FDR (false discovery rate) ≤ 0.001 and the absolute value of log_2_ RPKM ratio ≥ 1’ as the threshold to judge the significance of gene expression difference [55].

### Gene Ontology (GO) term analysis

Blast2GO program was used to classify unigenes to GO terms based on molecular function, biological processes and cellular components [56] for leaf and root tissues, at *p*-values < 0.05.

### Validation of RNA-Seq by qRT-PCR

qRT-PCR were performed to confirm a set of 32 differentially expressed genes (16 up-regulated and 16 down-regulated genes) randomly selected from each group of salt-inducible genes identified from RNA sequencing using a Chromo 4 real-time PCR detection system. Diluted cDNA was amplified using gene specific primers (S29 Table) and SYBR Green real-time PCR master mix (Toyobo). qRT-PCR was conducted with three biological replicates, and each sample was conducted at least in triplicate and normalized using *Tubulin* as an internal control [16].

## Results and Discussion

### Salt stress resistance assessment in the seedling stage of DXWR

**Fig 1:**
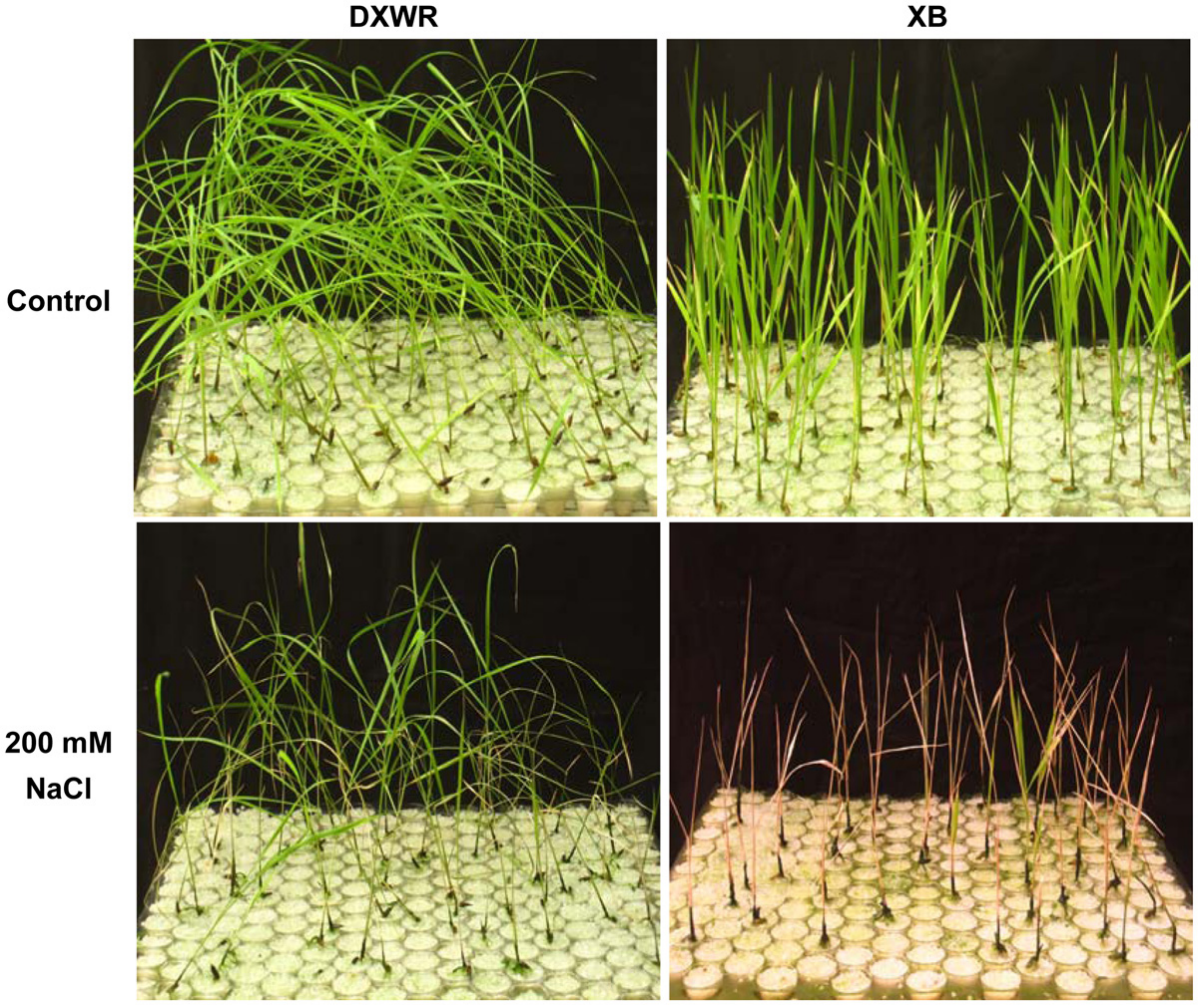
The seedlings of DXWR showed stronger salt resistance than the seedlings of XB.

To investigate the character of salt stress resistance in the DXWR seedlings, two-week-old seedlings of DXWR and Xieqingzao B (*O*. *sativa* L. ssp. *indica*, hereafter referred as XB), which is a representative maintainer line in hybrid rice breeding system in China [12] were exposed to the hydroponic solution with 200 mM NaCl for 12 days. When grown under normal, the DXWR and XB seedlings were all green and alive. After exposure to the salt stress, most DXWR seedlings remained green and showed continuous growth, whereas XB seedlings were all dead (Fig 1). The survival rate of the XB seedlings were 0%, but the survival rate of DXWR seedlings reached 80%. These results suggested that DXWR exhibited more salt-stress resistant than the cultivated rice XB at the seedling stage. In this study, 80% of DXWR seedlings were survival under 200 mM NaCl treatment for 12 days, in which the arrest of growth for many varieties of cultivated rice was generally found in previous studies [57–61]. Recent studies have proved that DXWR contained the genes involved in the improvement of salt tolerance in transgenic Arabidopsis [15, 16]. These results indicated that DXWR has the resistant to salt stress compared with the cultivated rice.

### Sequencing statistics

In order to understand the molecular mechanism of salt tolerance of DXWR, RNA-Seq was used to investigate gene expression responding to salt stress at the seedling stage. cDNA libraries were prepared from the leaves and roots of DXWR seedlings and subjected to RNA-Seq analysis on the Illumina HiSeq 2000 platform. A total of 46.8 million, 48.2 million, 43.6 million and 46.7 million high-quality 100-bp paired-end reads were obtained from the leaf and root transcriptome libraries of DXWR seedlings under the normal condition (control) and salt treatment (Table 1). The *O*. *sativa* ssp. *japonica* cv. Nipponbare genome has been completely sequenced through a map-based sequencing strategy [62]. Lu et al. isolated and completely sequenced 1888 putative full-length cDNA clones from wild rice *Oryza rufipogon* Griff. W1943 for comparative analysis between wild and cultivated rice species. Homology searching of these cDNA sequences revealed that > 96.8% of the wild rice cDNAs were matched to the cultivated rice Nipponbare genome sequence [63]. So in this study, we selected the Nipponbare genome for the references genome to map the reads. The alignment results showed that 70.41–74.63% of the total reads mapped to the reference genome and 58.84–63.34% to the gene regions (including 68.06–73.17% and 36.54–38.57% uniquely matched), respectively (Tables 1 and 2). We found that there was a significant difference in the percentage of reads mapping to the genome and gene regions, especially for the uniquely mapped reads. These results were similar with the previous study that about 72% of the total reads mapped to the genome and 46% to the gene regions (including 68% and 38% uniquely matched) for deep transcriptome sequencing of rhizome and aerial-shoot in *Sorghum propinquum* [28], suggesting that it might be result from the reads mapping to the intergenic regions and alternative mRNA splicing. The 25.37–29.59% of reads remained unmapped, attributing primarily to the gaps and diversity between DXWR and references genome sequences (Table 1). On average, at least 50% of more than 60% of the mapped genes were covered by the uniquely mapped reads, and only ~15% of the genes had gene coverage of 20% or lower (S1 Fig), indicating a good quality of the transcriptome. Previous studies have revealed that asian cultivated rice (*Oryza sativa*) was domesticated from the wild species *Oryza rufipogon* [64] and the sequence of wild rice W1943 had a very high similarity with those of Nipponbare [63]. But, several W1943 cDNAs that did not match to the Nipponbare genomic sequence might be located in the gap of genomic sequence or might be related to wild rice W1943-specific genes [63], suggesting that it has some limitations for using the Nipponbare genome to map the reads of *Oryza rufipogon*.

**Table 1:** Summary of Illumina transcriptome reads mapped to the reference genome.

Reads mapping	Reads number (%)			
	LCK	LS	RCK	RS
Total reads	46,784,432	48,221,828	43,588,908	46,715,548
Total base pairs	4,678,443,200	4,822,182,800	4,358,890,800	4,671,554,800
Total mapped reads	34,881,473 (74.56)	35,987,794 (74.63)	31,979,139 (73.37)	32,890,448 (70.41)
Perfect match	25,627,140 (54.78)	26,067,640 (54.06)	23,267,595 (53.38)	23,839,427 (51.03)
≤ 3 bp mismatch	9,254,333 (19.78)	9,920,154 (20.57)	8,711,544 (19.99)	9,051,021 (19.37)
Unique match	34,230,815 (73.17)	34,972,876 (72.52)	31,136,589 (71.43)	31,795,999 (68.06)
Multi-position match	650,658 (1.39)	1,014,918 (2.1)	842,550 (1.93)	1,094,449 (2.34)
Total unmapped reads	11,902,959 (25.44)	12,234,034 (25.37)	11,609,769 (26.63)	13,825,100 (29.59)

LCK, LS, RCK, and RS denote the control and salt treatment of the leaves and roots, respectively.

**Table 2:** Summary of Illumina transcriptome reads mapped to the reference genes.

Reads mapping	Reads number (%)			
	LCK	LS	RCK	RS
Total reads	46,784,432	48,221,828	43,588,908	46,715,548
Total base pairs	4,678,443,200	4,822,182,800	4,358,890,800	4,671,554,800
Total mapped reads	29,634,341 (63.34)	29,995,403 (62.20)	26,743,293 (61.35)	27,488,576 (58.84)
Perfect match	22,232,426 (47.52)	22,165,310 (45.97)	19,983,467 (45.85)	20,422,362 (43.72)
≤ 5 bp mismatch	7,401,915 (15.82)	7,830,093 (16.24)	6,759,826 (15.51)	7,066,214 (15.13)
Unique match	17,816,293 (38.08)	17,894,359 (37.11)	16,812,042 (38.57)	17,071,704 (36.54)
Multi-position match	11,818,048 (25.26)	12,101,044 (25.09)	9,931,251 (22.78)	10,416,872 (22.30)
Total unmapped reads	17,150,091 (36.66)	18,226,425 (37.80)	16,845,615 (38.65)	19,226,972 (41.16)

LCK, LS, RCK, and RS denote the control and salt treatment of the leaves and roots, respectively.

### Identification of differentially expressed genes (DEGs) by RNA-Seq

**Fig 2:**
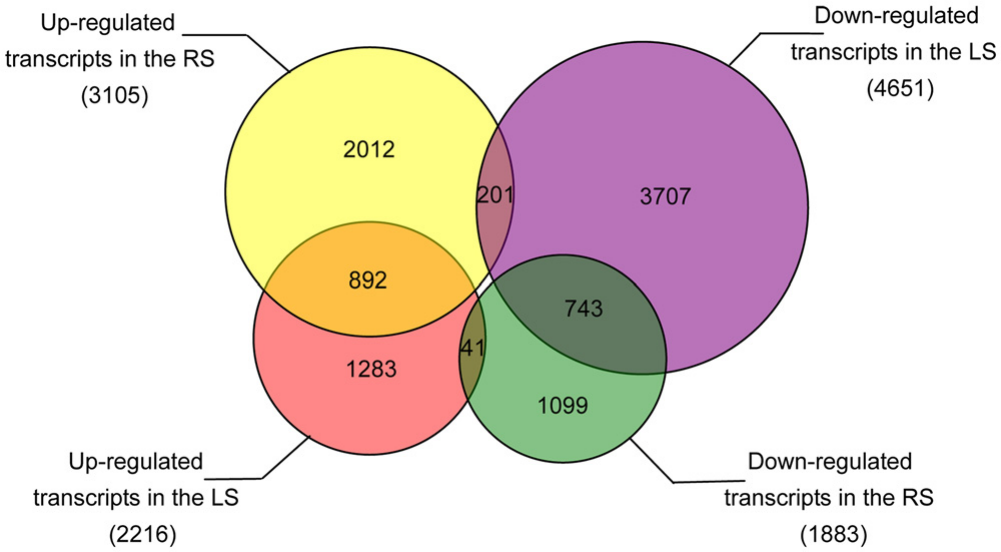
The number of up- and down-regulated transcripts in the LS and RS compared with the LCK and RCK. LS, leaves with salt treatment; RS, roots with salt treatment; LCK, leaves without salt treatment; RCK, roots without salt treatment.

Gene expression levels can be estimated from Illumina sequencing data based on the number of raw reads [65]. Putative DEGs from the treatment vs. control (LS vs. LCK and RS vs. RCK) were identified and a total of 2,216 transcripts showed up-regulation (S1 Table) and 4,651 transcripts showed down-regulation (S2 Table) in LS vs. LCK, whereas 3,105 transcripts showed up-regulation (S3 Table) and 1,883 transcripts showed down-regulation (S4 Table) in RS vs. RCK (Fig 2). Among the DEGs, 892 transcripts were up-regulated and 743 transcripts were down-regulated in both the LS vs. LCK and RS vs. RCK. Interestingly, 41 transcripts were up-regulated in LS vs. LCK but down-regulated in RS vs. RCK, and 201 transcripts were down-regulated in LS vs. LCK but up-regulated in RS vs. RCK. In these 201 transcripts, we detected that the expression of seven genes were dramatically changed (the absolute value of log_2_ RPKM ratio > 3), including LOC_Os03g37290, LOC_Os06g31800, LOC_Os09g13440, LOC_Os09g19229, LOC_Os10g13430, LOC_Os10g41040, and LOC_Os12g28177. But, so far little is known about their functions.

Among the DEGs, we found that a number of genes have been proved to be involved in responding to salt stress in the previous studies (Table 3). We also found that most of these genes are transcription factors (TFs), suggesting that TFs play critical roles in responding to salt stress via transcriptional regulation of the downstream genes responsible for plant tolerance to salt challenges.

**Table 3:** List of published salt resistant genes among the DEGs detected by RNA-Seq.

Gene name	Gene ID	Up or down (Log_2_ratio)		References
		LS vs. LCK	RS vs. RCK	
*ZFP179*	LOC_Os01g62190	up (3.67)	none	[61]
*ZFP182*	LOC_Os03g60560	up (5.65)	up (2.68)	[66]
*ZFP252*	LOC_Os12g39400	up (2.48)	none	[67]
*SNAC1*	LOC_Os03g60080	up (1.46)	up (1.72)	[68]
*SNAC2/OsNAC6*	LOC_Os01g66120	up (2.28)	up (1.21)	[69, 70]
*OsNAC5*	LOC_Os11g08210	up (1.26)	up (1.15)	[44]
*ONAC045*	LOC_Os11g03370	none	up (1.01)	[71]
*OsACA6*	LOC_Os04g51610	up (1.39)	none	[72]
*OsMYB2*	LOC_Os03g20090	up (1.39)	up (1.64)	[57]
*OsbZIP23*	LOC_Os02g52780	up (1.72)	none	[73]
*OsBIERF3*	LOC_Os02g43790	down (-1.13)	up (1.36)	[74]
*OsHKT1*	LOC_Os06g48810	none	down (-2.64)	[75]
*OsLEA3-2*	LOC_Os06g21910	up (12.63)	none	[76]
*RSOsPR10*	LOC_Os12g36830	up (1.91)	up (4.08)	[77]
*OsTZF1*	LOC_Os05g10670	up (1.15)	up (1.35)	[43]

Besides *ZFP179*, *ZFP182* and *ZFP252* which have been described for responding to salt stress by the previous studies, other zinc finger proteins (ZFPs) were also detected. For example, two C2H2-type ZFP genes, *ZOS3-12* (LOC_Os03g32230) which has been reported to be induced by nitrogen-starvation in rice [78] and *ZOS5-08* (LOC_Os05g37190) which has been proved to respond to grazing defoliation [79], indicating that ZFPs could play important roles in regulating various stress tolerance. A genome-wide annotation analysis of the CCCH-type ZFPs identified 67 genes in rice [80]. Apart from *OsTZF1* [43], we also detected other many CCCH-type ZFP genes (S5 Table), indicating that these CCCH-type ZFPs could be functional redundancy in salt-stress tolerance.

NAC (NAM, ATAF, and CUC) proteins are a class of plant-specific TFs, which contain a highly conserved N-terminal domain known as the NAC domain. Ooka et al. reported that 75 and 105 NAC genes were predicted in rice and *Arabidopsis* genomes, respectively [81]. Overexpression of four NAC domain genes, *SNAC1* [68], *SNAC2* [70], *OsNAC5* [82], and *ONAC045* [71], in rice conferred tolerance to salt stress. Besides these four genes described by previous studies, another 16 NAC domain genes were also found in our study (S6 Table). Our data combined with the previously determined functions of the NAC domain genes strongly suggested their important roles in salt-stress tolerance. Additionally, the 9 of 16 genes were down-regulated by salt stress in the leaves and/or roots of seedlings, and 6 of 16 genes were up-regulated by salt stress in the leaves or roots of seedlings. Interestingly, we also observed one gene, *ONAC066* (LOC_Os03g56580) that was down-regulated in the leaves, but up-regulated in the roots by salt stress. These results suggested that a number of NAC domain genes were involved in salinity tolerance, but there were different potential mechanisms of salt stress responses regulated by NAC domain genes.

MYB-type TFs play a diverse role in plant development and response to abiotic stress. MYB proteins contain one, two, or three imperfect repeats (51–53 amino acids) in their DNA-binding domain, and they are further classified into three subfamilies, type MYBR2R3, MYB-related, and type MYBR1R2R3, depending on the number of repeats in their MYB domains [83]. Recently, two R2R3-type MYB proteins (OsMYB2 [57] and OsMYB91 [7]) and one R1R2R3-type MYB protein (OsMYB3R-2) [84] were identified to be involved in response to salt stress. We found 62 MYB-type genes which were induced by salt treatment in the leaves and/or roots of DXWR seedlings besides *OsMYB2* (S7 Table). Among these genes, 47 genes were R2R3-type, 13 genes were MYB-related type, and 2 genes were R1R2R3-type. This result was consistent with that among the MYB proteins in plants, the MYB family with the two-repeat (R2R3) was the most common one and a large number of them were involved in responses of plants to environmental stress [85]. Moreover, a few MYB-related type genes were detected by our RNA-Seq analyses, and most of them (9 of 13) were up-regulated by salt stress, but so far, little is known about that the MYB-related type proteins are related with the response to salt stress. Our data indicated that the MYB-related type TFs could also participate in the response to salt stress, or there was another different potential mechanism of salt stress responses regulated by the MYB-related type TFs in DXWR seedlings compared with cultivated rice.

In plants, like other TF genes, members of the bZIP (basic leucine zipper) TF family also have been identified to regulate diverse plant-specific phenomena, including seed maturation and germination, floral induction and development, light signaling, and abiotic stress tolerance. 89 genes encoding bZIP TFs have been identified and characterized in the rice genome and they were classified into 10 subfamilies [86]. Recent studies have reported that *OsbZIP05* (*OSBZ8*) [87] and *OsbZIP23* [73] conferred salinity tolerance in rice. Besides *OsbZIP23*, we also found 27 salt-induced bZIP TFs distributed in 6 subfamilies (I, IV, V, VI, VII, and IX) (S8 Table). *OsbZIP38* (*LIP19*), *OsbZIP52* (*RISBZ5*), *OsbZIP87* (*OsOBF1*), *OsbZIP12*, *OsbZIP16*, and *OsbZIP45* have been identified to confer abiotic stress tolerance [88–92]. According to the previous studies and our RNA-Seq analyses, we observed that all these stress-inducible bZIP TFs were included in the above 6 subfamilies, suggesting that the bZIP TFs of these 6 subfamilies may play more important roles in responding to abiotic stress compared with the other subfamilies. Furthermore, recent studies have proved that *OsbZIP12* [93], *OsbZIP23* [73], and *OsbZIP45* [92] were involved in not only drought tolerance but also salinity tolerance. *OsbZIP16* also has been identified to positively regulate drought resistance in rice [91]. *OsbZIP71* was shown to play an important role in ABA-mediated drought and salt tolerance in rice [41]. Our data combined with the previous studies strongly suggested that there could be the similar potential mechanisms regulated by the bZIP TFs between the salt and drought stress responses.

In plant kingdom, AP2/ERF (APETALA2/ethylene response factor) is a large family of TFs. To date, 163 AP2/ERF-type TFs were identified in rice and divided into four subfamilies [94]. The AP2/ERF-type TFs have been shown to be highly involved in the salt stress tolerance mechanisms [3, 74, 95–97]. Our RNA-Seq analyses showed that 41 AP2/ERF-type TF genes were up-regulated or down-regulated by salt stress in the leaves and/or roots of seedlings. Among these genes, eight genes belonged to the AP2 subfamily; twelve genes belonged to the DREB subfamily, seventeen genes belonged to the ERF subfamily, and four genes belonged to the RAV subfamily (S9 Table). Although a few AP2/ERF genes were identified to be related with the response to salt stress, they all belonged to the DREB and ERF subfamilies and little is known about whether the TFs of AP2 and RAV subfamilies participate in the salt-stress response. We found eight AP2 genes and four RAV genes, suggesting that there could be some unknown molecular mechanisms of salt stress responses regulated by the TFs of AP2 and RAV subfamilies in rice or some special mechanism of salt stress responses regulated by AP2/ERF-type TFs in DXWR seedlings compared with cultivated rice. Additionally, we found all eight AP2 genes were down-regulated by salt stress and except of one gene (LOC_Os11g03540) was down-regulated in both the leaves and roots, all the other genes were down-regulated only in leaves. These results suggested that the AP2-type TFs could mainly negatively regulate salt stress responses in the leaves of DXWR seedlings.

To further identify the novel TFs involved in the salt stress tolerance in our study, the striking up-regulated TF genes were selected (the value of log_2_ RPKM ratio > 3). In the LS vs. LCK, we detected 20 TF genes, including two bHLH (basic helix-loop-helix) genes, seven ZFP genes, and two WRKY genes (S10 Table). In the RS vs. RCK, we found 26 TF genes, including nine ZFP genes, five WRKY genes, and four bHLH genes (S11 Table). Among these genes, four genes were up-regulated both in the LS vs. LCK and the RS vs. RCK, including *OsWRKY42* (LOC_Os02g26430), two Homeodomain-leucine zipper TF genes (LOC_Os02g43330 and LOC_Os04g45810), and one GRAS family TF gene (LOC_Os11g04400). To date, expect *OsWRKY42* [42] and *OsbHLH133* (LOC_Os12g32400) [98] which have been studied for their functions, little was known about the functions for the other genes in rice. Research on salt regulatory TFs has mainly focused on single factors and linear pathways. However, emerging findings increasingly suggest integration of the TFs in dynamic network hubs as well as interaction and competition of pathways manifesting complexity of molecular links in stress adaptation. After salt stress, the plant hormone biosynthesis was changed dynamically and then Ca^2+^, ROS (reactive oxygen species), and hormone signaling cascades were activated. At the next step, the global transcriptional profile of the plant (including TF families, such as bZIP, NAC, AP2/ERF, WRKY, bHLH and MYB) could be altered and regulated many salt stress responsive genes expression. Ultimately, these early signaling pathways result in expression and activation of cellular detoxification mechanisms, including HKT, NHX, and the SOS Na^+^ transport mechanisms as well as osmotic protection strategies [40]. Furthermore, within this hierarchical network, cellular stress responses might be fine tuned by interaction and competition of TFs that regulate sub-clusters of the stress transcriptome. In the hierarchical network of TFs for stress adaptation, bZIP TFs could be in the upstream position and other TFs might have functions in regulating sub-networks of adaptation to salt stress [32].

Besides a large number of TFs, two HKT (high-affinity potassium transporter) genes, *OsHKT1* and *OsHKT7* were detected by our RNA-Seq analyses. Consisting with the observation that the *OsHKT1* transcript was significantly down-regulated in salt-tolerant rice under NaCl stress [75], we observed that *OsHKT1* was down-regulated by salt stress in the roots of DXWR seedlings. There is little known about that *OsHKT7* contributes to salt tolerance in rice, but its homologue, *TmHKT7* in durum wheat (T*riticum turgidum* subsp. *durum*) was identified to be involved in salt stress response [99]. We found that *OsHKT7* was down-regulated by salt stress both in the leaves and roots, suggesting that *OsHKT1* and *OsHKT7* could act redundantly to negatively regulate salt stress response.

To confirm the validity of the DEG data, quantitative RT-PCR was performed to investigate the expression patterns of 32 randomly selected genes under the same conditions. We compared the results obtained from quantitative RT-PCR with the gene expression profiles from RNA-Seq analysis. Expression trends were consistent for all transcripts in both analyses, with a correlation coefficient of *R*^*2*^ = 0.8961 (S2 Fig).

### Functional classification by Gene Ontology (GO)

**Fig 3:**
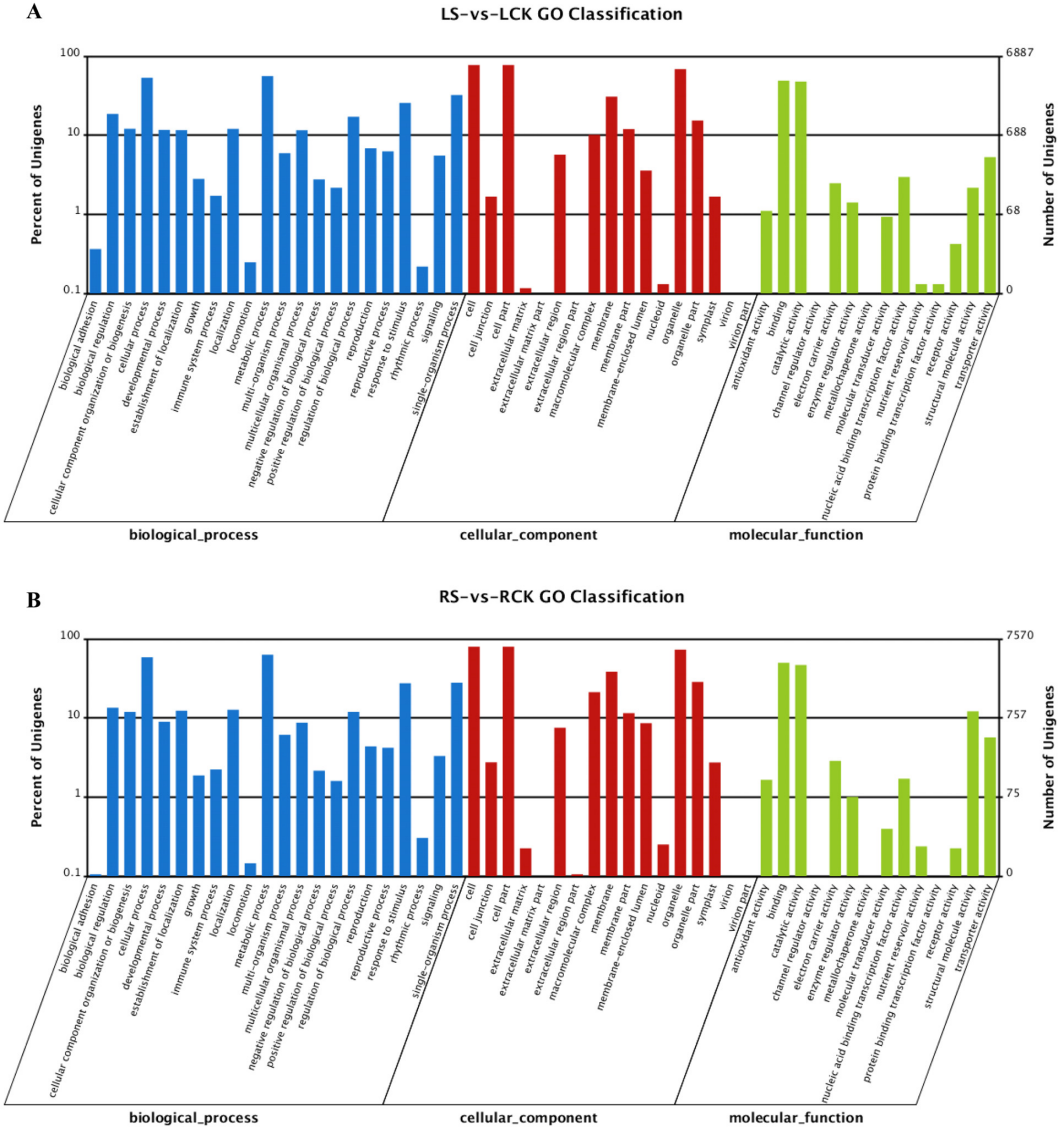
Gene ontology (GO) classification of the unigenes from the LS-vs-LCK (A) and RS-vs-RCK (B).

Web Gene Ontology Annotation Plot (WEGO) software [100] was used to perform the GO classifications and to draw the GO tree to classify the up- and down-regulated transcripts into putative functional groups for the LS vs. LCK and RS vs. RCK. A total of 15,636 and 17,937 transcripts were assigned GO terms for the DEGs in the LS vs. LCK and RS vs. RCK, respectively. Among the 15,636 transcripts from the LS vs. LCK (Fig 3A), there were 5,633 transcripts at the cellular level, 5,068 transcripts at the molecular level and 4,935 transcripts at the biological level. Among the 17,937 transcripts from the RS vs. RCK (Fig 3B), there were 6,302 transcripts at the cellular level, 5,907 transcripts at the molecular level and 5,728 transcripts at the biological level. In the biological process category, cellular process and metabolic process were the most highly represented groups, suggesting that extensive metabolic activities were taking place both in the leaves and roots of DXWR seedlings with salt treatment. Within the cellular component category, transcripts that corresponded to cell, cell parts and cell organelles were the most abundant. Binding and catalytic activities were the most abundant groups within the molecular functional category.

We further identified GO terms in the biological process category that were over-represented (*P* < 0.05) in DEGs of LS vs. LCK and RS vs. RCK, respectively (S12 and S13 Tables). These GO terms served as indicators of significantly different biological processes underlying salt-stress responses in the leaves and roots. GO terms such as metabolic process, catabolic process, and response to stimulus (Table 4) were enriched in both sets of transcripts from the two tissues, suggesting that the same biological processes were required to maintain the tissues activities during salt treatment. However, some striking differences were found between the two sets of enriched GO terms. In particular, GO terms related to cell cycle process and DNA reproductive process were highly enriched in the DEGs of LS vs. LCK, suggesting that salt stress may affect the reproduction of the plant cell and the plant may utilize this effect to resist the salt stress in the leaves of seedlings. For the RS vs. RCK, there were a large number of DEGs involved in transport, response to metal ion, response to abiotic stimulus, and regulation of reactive oxygen species metabolic process. This result indicated that the roots could be the main tissue for the plant to resist the salt stress and maintaining a low cytosolic Na^+^ concentration could be the most important way to improve salt-stress tolerance for the plant [40]. We also further identified GO terms in the cellular component and molecular function categories that were over-represented (*P* < 0.05) in DEGs of LS vs. LCK and RS vs. RCK, respectively (S14–S17 Tables). In the cellular component category, cell periphery and extracellular region were enriched in both sets of transcripts from the two tissues (S18 Table). This indicated that that salt stress affected the cell-cell interactions. The cytoplasmic membrane-bounded vesicle was also enriched in the two tissues, indicating an involvement of endosomal transport proteins, including NHXs, in plant salt tolerance, by transporting K+ into vacuoles to control organelle pH and ion homeostasis [101]. For the molecular function category, endopeptidase regulator activity was enriched in the two tissues (S19 Table). This suggested that some stress responsive genes expression might be regulated by posttranslational modification to cope with salinity stress. The peptidase regulator activity and carbohydrate derivative binding were enriched and this was consistent with the previous studies that the accumulation of organic osmolytes, such as sugar alcohols, proline, and polyamines, plays a key role in maintaining the low intracellular osmotic potential of plants and in preventing the harmful effects of salt stress [102, 103].

**Table 4:** The significant GO terms of DEGs for the biological process category both in the LS vs. LCK and RS vs. RCK.

GO term	GO term annotation
GO:0006022	aminoglycan metabolic process
GO:0006026	aminoglycan catabolic process
GO:0006030	chitin metabolic process
GO:0006032	chitin catabolic process
GO:0010035	response to inorganic substance
GO:0010466	negative regulation of peptidase activity
GO:0022607	cellular component assembly
GO:0034622	cellular macromolecular complex assembly
GO:0043933	macromolecular complex subunit organization
GO:0046348	amino sugar catabolic process
GO:0050896	response to stimulus
GO:0051346	negative regulation of hydrolase activity
GO:0052547	regulation of peptidase activity
GO:0065003	macromolecular complex assembly
GO:1901072	glucosamine-containing compound catabolic process

Especially, we detected some enriched GO terms for the biological process category in LS vs. LCK, including chromatin assembly or disassembly, nucleosome assembly, histone phosphorylation and histone H3-K9 methylation, which belonged to epigenetic processes and were related to transcription. Efficiency of gene expression is highly influenced by chromatin structure that might be modulated epigenetically by processes as DNA methylation and posttranslational modifications of histones. The histone-mediated structure of nucleosomes in the chromatin might be modified and thus influence nucleosome density, binding efficiency of TFs, and transcriptional activity [104]. Several studies have shown that chromatin modification contribute to plant environmental adaptation [105, 106]. However, in contrast to the detailed knowledge on influences of epigenetic mechanisms on developmental processes, information on epigenetic regulation of abiotic stress resistance is still rare.

### Kyoto Encyclopedia of Genes and Genomes (KEGG) pathway mapping

**Fig 4:**
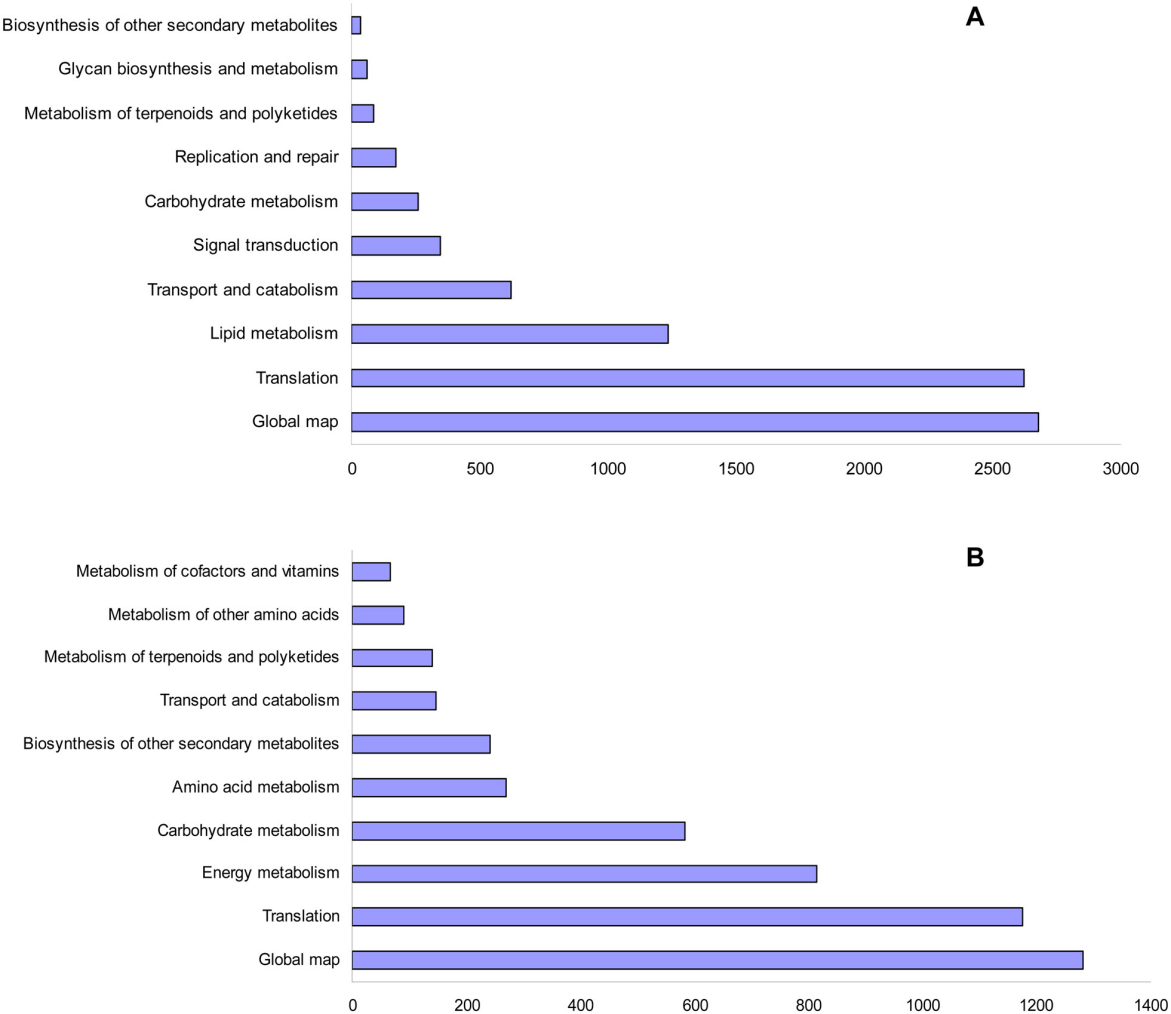
KEGG pathway assignments in the LS vs. LCK (A) and RS vs. RCK (B). The represented categories (Q-value≤ 0.05) and the number of transcripts predicted to belong to each category are shown.

To identify metabolic pathways in which DEGs were involved and enriched, pathway-based analysis was performed using the KEGG pathway database [107]. As a result, 4,131 of 6,876 in the LS vs. LCK and 3,069of 4,988 in the RS vs. RCK were classified into 20 functional categories. Then they were classified into 125 and 126 subcategories, respectively. We further identified over-represented KEGG Orthology (KO) terms (*Q*-value < 0.05), and classified them into 10 categories, respectively (Fig 4). As shown in Fig 4, these transcripts belonged mainly to the following KEGG pathways both in the LS vs. LCK and RS vs. RCK: Biosynthesis of other secondary metabolites, carbohydrate metabolism, global map, metabolism of terpenoids and polyketides, translation, and transport and catabolism. Then they were further classified into 19 and 27 subcategories (S20 and S21 Tables). Among these subcategories, we found that five subcategories, metabolic pathways, biosynthesis of secondary metabolites, amino sugar and nucleotide sugar metabolism, RNA transport, and mRNA surveillance pathway were over-represented both in the LS vs. LCK and RS vs. RCK (S3 Fig). Furthermore, RNA transport and mRNA surveillance pathways were related to transcription, indicating that these pathways might have a modulating impact on the regulation of salt-inducible gene expression. In contrast, KO terms related to endocytosis, ether lipid metabolism, and glycerophospholipid metabolism were exclusively enriched in the LS vs. LCK. This result suggested that there could be considerable differences of biochemical and physiological processes between the leaves and roots of seedlings during salt stress. These annotations provide a valuable resource for investigating specific processes, functions, and pathways of salt-stress responses in different tissues.

### Comparative analysis of the DEGs in our RNA-Seq with those previously identified by RNA-Seq in cultivated rice Nipponbare

Cultivated rice is considered to have been domesticated from wild rice thousands of years ago. However, in the long-term domestication, cultivated rice lost various valuable traits with regard to tolerance to cold, drought and salinity which derived from wild rice [9]. To investigate the similarities and differences of salt stress responsive mechanisms between cultivated rice and wild rice, we compared our RNA-Seq data with the data of the previous study which used RNA-Seq to investigate the DEGs in the shoots and roots of Nipponbare seedlings with 1 h of salinity stress compared with normal rice shoots and roots [30]. According to the above criteria: false discovery rate (FDR) ≤ 0.001 and the absolute value of log_2_ RPKM ratio ≥ 1, the previous study showed that a total of 665 genes were up-regulation (S22 Table) and 296 genes were down-regulation (S23 Table) in shoots, whereas 1,365 genes were up-regulation (S24 Table) and 1,425 genes were down-regulation (S25 Table) in roots. Among the DEGs, 352 genes were up-regulated and 76 genes were down-regulated in both the shoots and roots.

**Fig 5:**
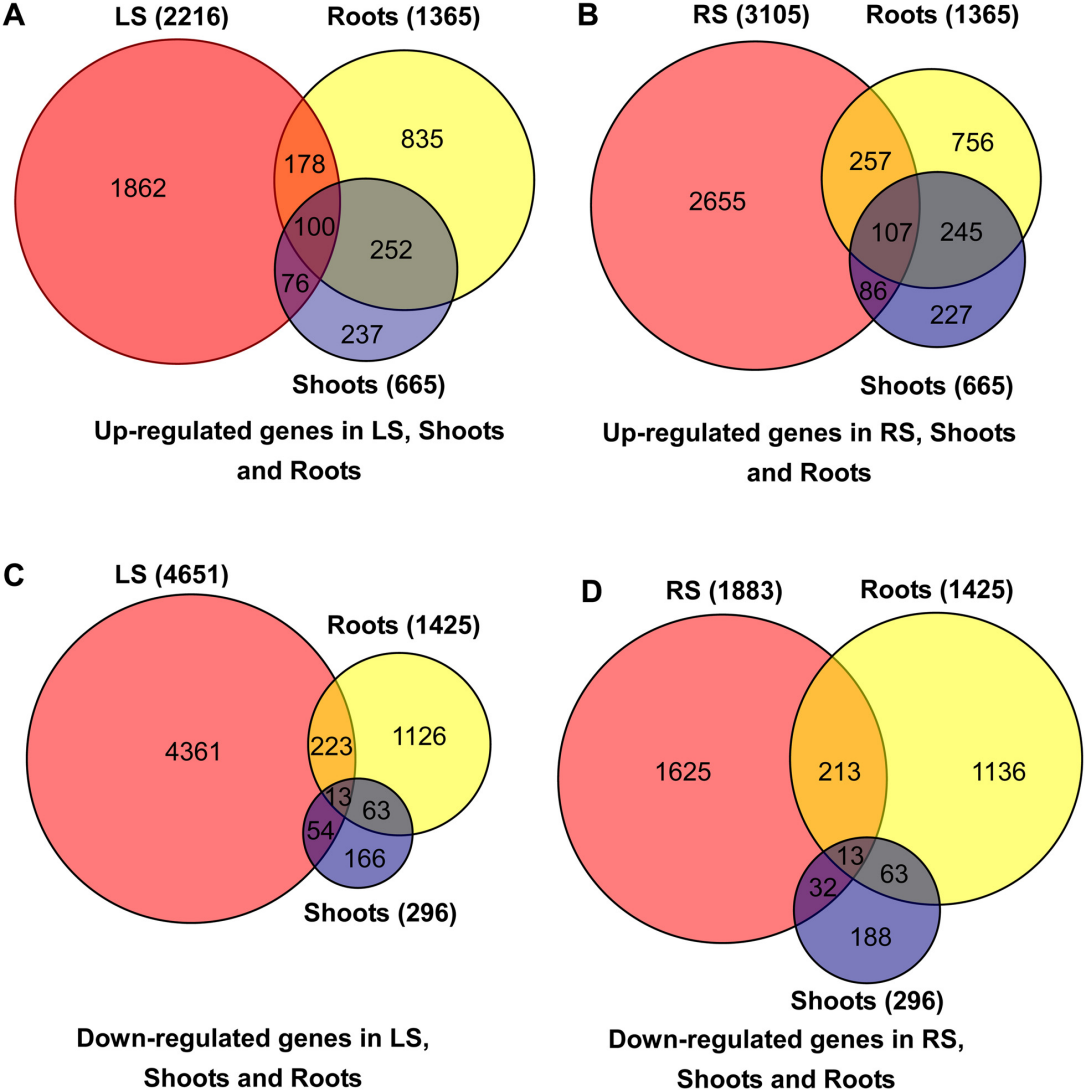
The number of up- and down-regulated genes in the LS (LS vs. LCK), RS (RS vs. RCK), shoots, and roots. (A), up-regulated genes in the LS, shoots, and roots; (B), up-regulated genes in the RS, shoots, and roots; (C), down-regulated genes in the LS, shoots, and roots; (D), down-regulated genes in the RS, shoots, and roots.

Comparing our data with the previous study, we found that 176 genes were up-regulated in both the LS vs. LCK (for convenient description, LS vs. LCK was named as LS) and shoots, 278 genes were up-regulated in both the LS and roots, and 100 genes were all up-regulated in the LS, shoots and roots (Fig 5A). We also found that 67 genes were down-regulated in both the LS and shoots, 236 genes were down-regulated in both the LS and roots, and 13 genes were all down-regulated in the LS, shoots and roots (Fig 5C). We observed that 193 genes were up-regulated in both the RS vs. RCK (for convenient description, RS vs. RCK was named as RS) and shoots, 364 genes were up-regulated in both RS and roots, and 107 genes were all up-regulated in RS, shoots and roots (Fig 5B). We also observed that 45 genes were down-regulated in both the RS and shoots, 226 genes were down-regulated in both the RS and roots, and 13 genes were all down-regulated in the RS, shoots and roots (Fig 5D).

Furthermore, we observed that 57 genes and 8 genes were all up-regulated and down-regulated in the LS, RS, shoots, and roots, respectively (S26 and S27 Tables). Among the 57 up-regulated genes, one LEA (late embryogenesis abundant) gene (LOC_Os01g21250) was found and the previous studies have proved that the LEA genes, *OsLEA3-2* [76] and *JcLEA* [108] conferred salinity tolerance in the transgenic *Arabidopsis* plants, respectively. Our data combined with the previous studies strongly suggested that the plants could have a common molecular mechanism regulated by LEA proteins to respond to salt stress in different tissues. We also found four WRKY TFs, WRKY24, WRKY28, WRKY70, and WRKY113 among the 57 up-regulated genes. This result is consistent with the previous observations that WRKY-type TFs play important roles in the adaptation to abiotic stresses [109–111]. Two salt-responsive genes (LOC_Os01g64360 and LOC_Os02g52670) which belonged to MYB TF family and AP2/ERF TF family respectively were also observed not only in DXWR but also in cultivated rice Nipponbare, indicating that the salt-stress response pathway regulated by MYB and AP2/ERF TFs in cultivated rice may originate from wild rice.

Plants respond to abiotoc stress through a complexity of signaling pathways, and the dephosphorylation mediated by protein phosphatase (PP) is an important event in this process. In the rice genome, 90 PP2C were identified and classified into 11 groups [112]. Recent studies have reported that two PP2C genes, *OsPP18* [113] and *OsPP108* [114] were essential for plant tolerance to several abiotic stresses, such as drought, salt, mannitol, and oxidative stresses. Among the 57 up-regulated genes, we also found two PP2C genes (LOC_Os03g16170 and LOC_Os09g15670). These results suggested that many PP2C genes could act redundantly in the phosphorylation cycles to make the phosphorylated proteins to switch rapidly from one state to another, which allows rice to respond to stress stimuli rapidly and accurately. 13 TPP (trehalose-6-phosphate phosphatase) genes and 14 TPS (trehalose-6-phosphate synthase) have been identified in the rice and one TPP gene, *OsTPP1* [115] and one TPS gene, *OsTPS1* [116] have been found that their overexpression in rice enhanced tolerance to abiotic stress by activating stress tolerance related genes. Besides *OsTPP5* (LOC_Os03g26910) which was found in the 57 up-regulated genes, we also found three TPP genes (*OsTPP6*, *OsTPP7*, and *OsTPP8*), and four TPS genes (*OsTPS4*, *OsTPS6*, *OsTPS9*, and *OsTPS10*) in DXWR and Nipponbare, but not all in the LS, RS, shoots, and roots. Moreover, one TPS gene, *OsTPS8* was only found in DXWR but not in Nipponbare. These results suggested that TPP genes and TPS genes could act together to confer stress tolerance through activating stress responsive genes, but different tissues had their own specific salt-stress response pathway regulated by TPP and TPS genes.

Two RLCK (receptor-like cytoplasmic kinase) genes, *OsRLCK167* (LOC_Os04g56060) and *OsRLCK253* (LOC_Os08g28710) were observed in the 8 down-regulated genes and 57 up-regulated genes, respectively. This result was consistent with the previous study that *OsRLCK167* was down-regulated and *OsRLCK253* was up-regulated under salt stress in rice seedlings [117]. To date, however, the molecular mechanism of salt-stress responses regulated by RLCK genes remained unknown. According to our RNA-Seq analyses and previous researches, we supposed that these two genes could play antagonistic roles in responding to salt stress. Taken together, the number of salt-inducible genes in DXWR is much more than that of cultivated rice Nipponbare, whether it was the number of up-regulated genes or down-regulated genes, suggesting that DXWR may have more complex molecular mechanisms and signaling pathways than the cultivated rice Nipponbare to preferably adapt to salt stress. Meanwhile, we should realize that there could be limitations in comparison of RNA-Seq results, due to technical differences, such as differences in depth of RNA-Seq experiments and differences in read lengths and mapping. Therefore, the differences between DXWR and Nipponbare in salt-stress responses need to be further analyzed in future.

### DEGs mapped to the previously identified salt stress related QTL intervals

Map-based cloning is one approach that may be used for the identification of genes underlying QTLs. Several QTLs associated with yield [118], cold tolerance [13], brown planthopper resistance [11], and drought tolerance [9] have been identified in DXWR. Based on the Gramene QTL database (www.gramene.org/db/), a total of 17 QTLs related to salt tolerance in rice have been identified. We located 276 genes differentially regulated by salt stress on 7 of these identified QTL intervals. Among them, the QTLs *qST1*, *qSDS-6*, and *qRNC-9* had the greatest number of co-localized DEGs with 82, 41, and 41 genes, respectively (S28 Table).

The QTL *qST1* is the most important QTL related to salt stress tolerance in rice at seedling stage [119]. 82 DEGs were co-localized on the *qST1* interval. Among them, many TFs were found, including two NAC genes, *ONAC048* and *ONAC068*; three WRKY genes, *WRKY22*, *WRKY24*, and *WRKY108*; three C2H2-type ZFP genes, *ZOS1-14*, *ZOS1-15*, and *ZOS1-17*; three bZIP genes, *OsbZIP10*, *OsbZIP11*, and *OsbZIP12*; one MYB gene (LOC_Os01g64360); one C3HC4-type ZFP gene ((LOC_Os01g64620); one AP2/ERF gene (LOC_Os01g64790). We also found one PP2C gene ((LOC_Os01g62760) and the *P5CS1* gene which encodes for a bifunctional enzyme that catalyzes the rate limiting reaction in proline biosynthesis in living organisms and has been proved to be involved in abiotic stress tolerance [120].

The major QTL associated with survival days of seedlings under salt stress, designated as *qSDS-6* [121], was co-localized with 41 DEGs, including one GATA-type ZFP gene (LOC_Os06g37450) and a VQ gene (LOC_Os06g40090) which has been reported to be involved in response to abiotic stresses [122]. 41DEGs co-localized on a major QTL *qRNC-9*, which included four TF genes, two bHLH (basic helix-loop-helix) genes (LOC_Os09g24490 and LOC_Os09g28210), one WRKY gene WRKY62, and one C3HC4-type ZFP gene (LOC_Os09g29310). In addition, we also found one annexin gene (LOC_Os09g27990) and the rice annexin genes have been found to be regulated in seedlings stage by various abiotic stressors including salinity, drought, heat and cold [123]. By combining further functional identification and QTL fine mapping, the co-localized DEGs detected in this RAN-Seq analysis may provide the basic for gene cloning and elucidation of the common molecular mechanisms responsible for salt tolerance between in the DXWR and rice.

## Conclusions

DXWR has several desired agronomic traits, and therefore it is considered as an important genetic resource for rice breeding. In this study, we used RNA-Seq platform to systematically investigate the salinity stress-inducible transcripts of leaves and roots at the seedling stage of DXWR. Identified DEGs with contrasting expression patterns between leaves and roots in response to salt stress are excellent targets for further functional studies to understand more specific molecular mechanisms of salt tolerance. Furthermore, this data set, compared with the previous RNA-Seq analysis and QTLs for salt tolerance in rice, provides clues for candidate transcripts and more complete information that are essential for future studies into the specific molecular mechanisms of salt-stress responses in DXWR. Further experiments are in progress towards functional validation of putative candidate genes to provide genetic resources for the improvement of salt-stress tolerance in rice.

## Supporting Information

S1 Fig Distribution of genes coverage in the leaves and roots of DXWR seedlings with or without salt treatment, respectively. (PDF)

S2 Fig Comparison of the expression of 32 randomly selected genes using RNA-Seq and qRT-PCR. (PDF)

S3 Fig The subcategories of over-represented KO terms (*Q*-value < 0.05) in the LS vs. LCK and RS vs. RCK. (PDF)

S1 Table List of up-regulated genes in LS vs. LCK. (XLS)

S2 Table List of down-regulated genes in LS vs. LCK. (XLS)

S3 Table List of up-regulated genes in RS vs. RCK. (XLS)

S4 Table List of down-regulated genes in RS vs. RCK. (XLS)

S5 Table List of CCCH-type ZFP genes among the DEGs detected by RNA-Seq. (PDF)

S6 Table List of NAC-type genes among the DEGs detected by RNA-Seq. (PDF)

S7 Table List of MYB-type TF genes among the DEGs detected by RNA-Seq. (PDF)

S8 Table List of bZIP TF genes among the DEGs detected by RNA-Seq. (PDF)

S9 Table List of AP2/ERF TF genes among the DEGs detected by RNA-Seq. (PDF)

S10 Table List of TF genes among the significant up-regulated DEGs detected in LS vs. LCK. (PDF)

S11 Table List of TF genes among the significant up-regulated DEGs detected in RS vs. RCK. (PDF)

S12 Table Significant GO terms of DEGs in the biological process category for LS vs. LCK. (PDF)

S13 Table Significant GO terms of DEGs in the biological process category for RS vs. RCK. (PDF)

S14 Table Significant GO terms of DEGs in the cellular component category for LS vs. LCK. (PDF)

S15 Table Significant GO terms of DEGs in the cellular component category for RS vs. RCK. (PDF)

S16 Table Significant GO terms of DEGs in molecular function category for LS vs. LCK. (PDF)

S17 Table Significant GO terms of DEGs in molecular function category for RS vs. RCK. (PDF)

S18 Table The significant GO terms of DEGs for the cellular component category both in the LS vs. LCK and RS vs. RCK. (PDF)

S19 Table The significant GO terms of DEGs for the molecular function category both in the LS vs. LCK and RS vs. RCK. (PDF)

S20 Table Significant KO terms of DEGs in the LS vs. LCK (*Q*-value < 0.05). (PDF)

S21 Table Significant KO terms of DEGs in the RS vs. RCK (*Q*-value < 0.05). (PDF)

S22 Table List of up-regulated genes in shoots [30]. (XLS)

S23 Table List of down-regulated genes in shoots [30]. (XLS)

S24 Table List of up-regulated genes in roots [30]. (XLS)

S25 Table List of down-regulated genes in roots [30]. (XLS)

S26 Table List of the same up-regulated genes among the LS, RS, shoots, and roots. (PDF)

S27 Table List of the same down-regulated genes among the LS, RS, shoots, and roots. (PDF)

S28 Table Co-localization of DEGs onto the previous detected QTLs responsible for salt treatment in rice. (XLS)

S29 Table Primers used for qRT-PCR analysis of differentially expressed genes. (PDF)

## Abbreviations

AP2: APETALA2
bHLH: basic helix-loop-helix
bZIP: basic leucine zipper
DEGs: differentially expressed genes
DXWR: Dongxiang wild rice
ERF: ethylene response factor
FDR: false discovery rate
GO: Gene Ontology
HKT: high-affinity potassium transporter;
KEGG: Kyoto Encyclopedia of Genes and Genomes
KO: KEGG Orthology
LEA: late embryogenesis abundant
NAC: NAM, ATAF, and CUC
PP: protein phosphatase
RPKM: reads per kilobase per million reads
QTLs: quantitative trait loci
RLCK: receptor-like cytoplasmic kinase
TFs: transcription factors
TPP: trehalose-6-phosphate phosphatase
WEGO: Web Gene Ontology Annotation Plot
XB: Xieqingzao B
ZFPs: zinc finger proteins
