# Supplementary material for: Transcriptome Analysis of Salt Stress Responsiveness in the Seedlings of Dongxiang Wild Rice (Oryza rufipogon Griff.)
Source: PLoS One. 2016 Jan 11;11(1):e0146242. doi: 10.1371/journal.pone.0146242 (PMC4709063; doi:10.1371/journal.pone.0146242)
Supplement: S1 Fig — (PDF) [file pone.0146242.s001.pdf]

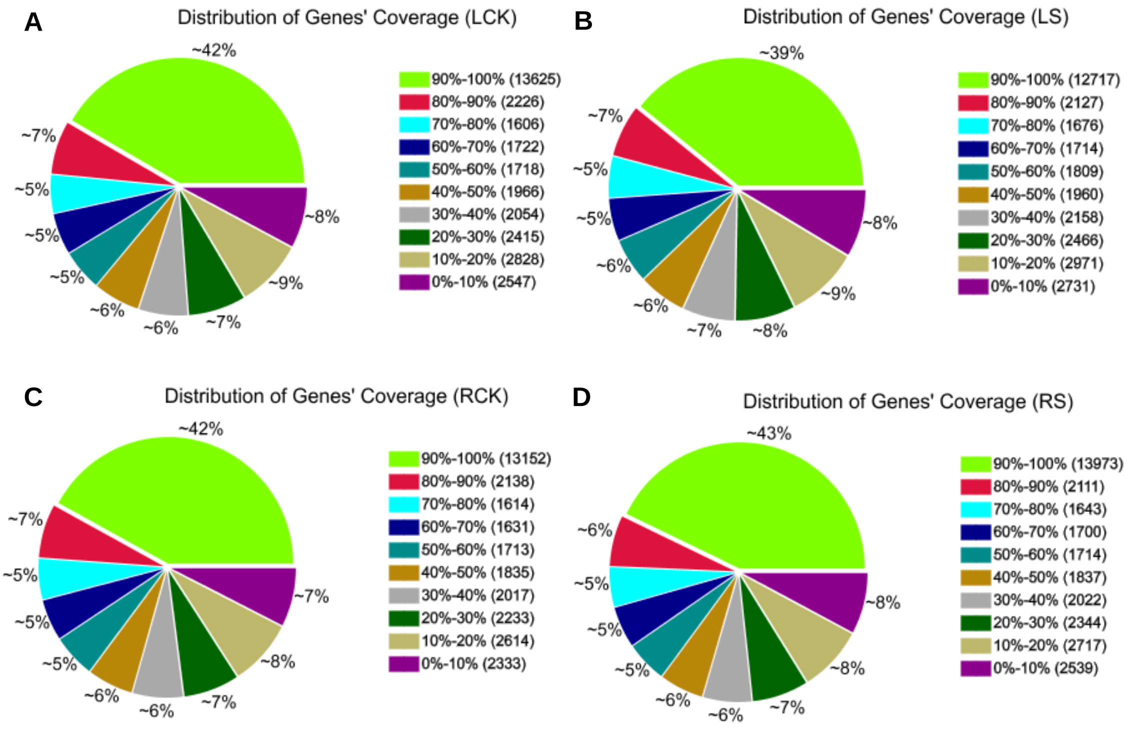

S1 Fig. Distribution of genes coverage in the leaves and roots of DXWR seedlings with or without salt treatment, respectively. **A** leaves without salt treatment (LCK). **B** leaves with salt treatment (LS). **C** roots without salt treatment (RCK). **D** roots with salt treatment (RS). Gene coverage is the percentage of a gene that is covered by reads and defined as the ratio of the number of bases in a gene covered by uniquely mapped reads to the number of total bases in the gene. The pie graph demonstrates the detailed percentage of the different gene coverage listing on the left of the pie graph.
