# Supplementary material for: Transcriptome Analysis of Salt Stress Responsiveness in the Seedlings of Dongxiang Wild Rice (Oryza rufipogon Griff.)
Source: PLoS One. 2016 Jan 11;11(1):e0146242. doi: 10.1371/journal.pone.0146242 (PMC4709063; doi:10.1371/journal.pone.0146242)
Supplement: S2 Fig — (PDF) [file pone.0146242.s002.pdf]

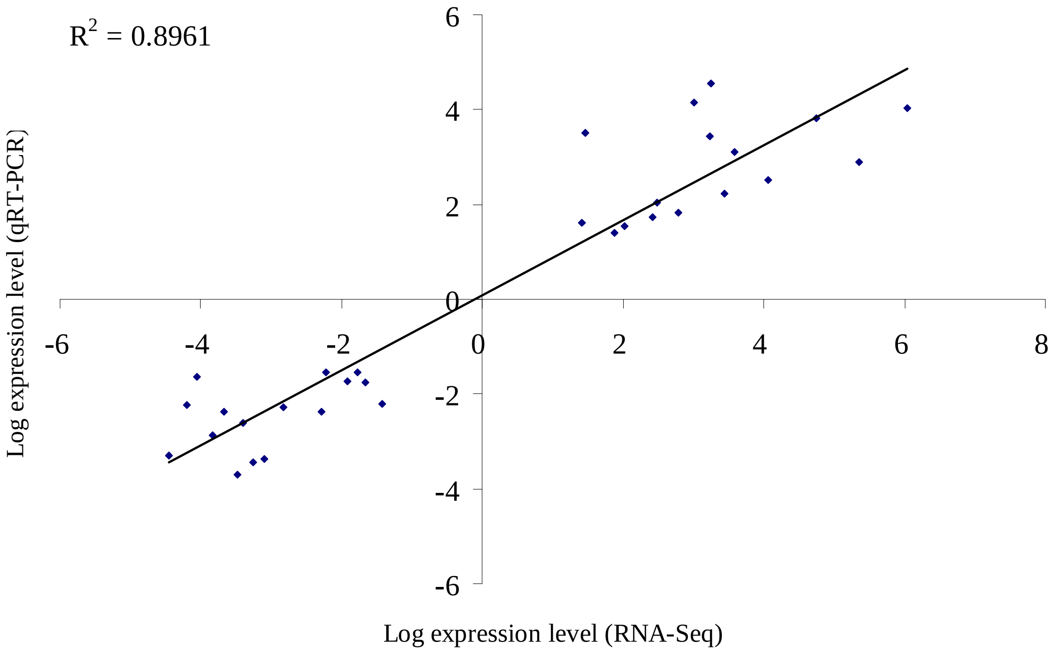

S2 Fig. Comparison of the expression of 32 randomly selected genes using RNA-Seq and qRT-PCR. The gene expression values were transformed to $\log_2$ scale. The RNA-Seq data $\log_2$-value (*X*-axis) was plotted against the qRT-PCR $\log_2$-value (*Y*-axis).
