# Supplementary figures and images for: Transcriptome Analysis of Salt Stress Responsiveness in the Seedlings of Dongxiang Wild Rice (Oryza rufipogon Griff.)
Source: PLoS One. 2016 Jan 11;11(1):e0146242. doi: 10.1371/journal.pone.0146242 (PMC4709063; doi:10.1371/journal.pone.0146242)

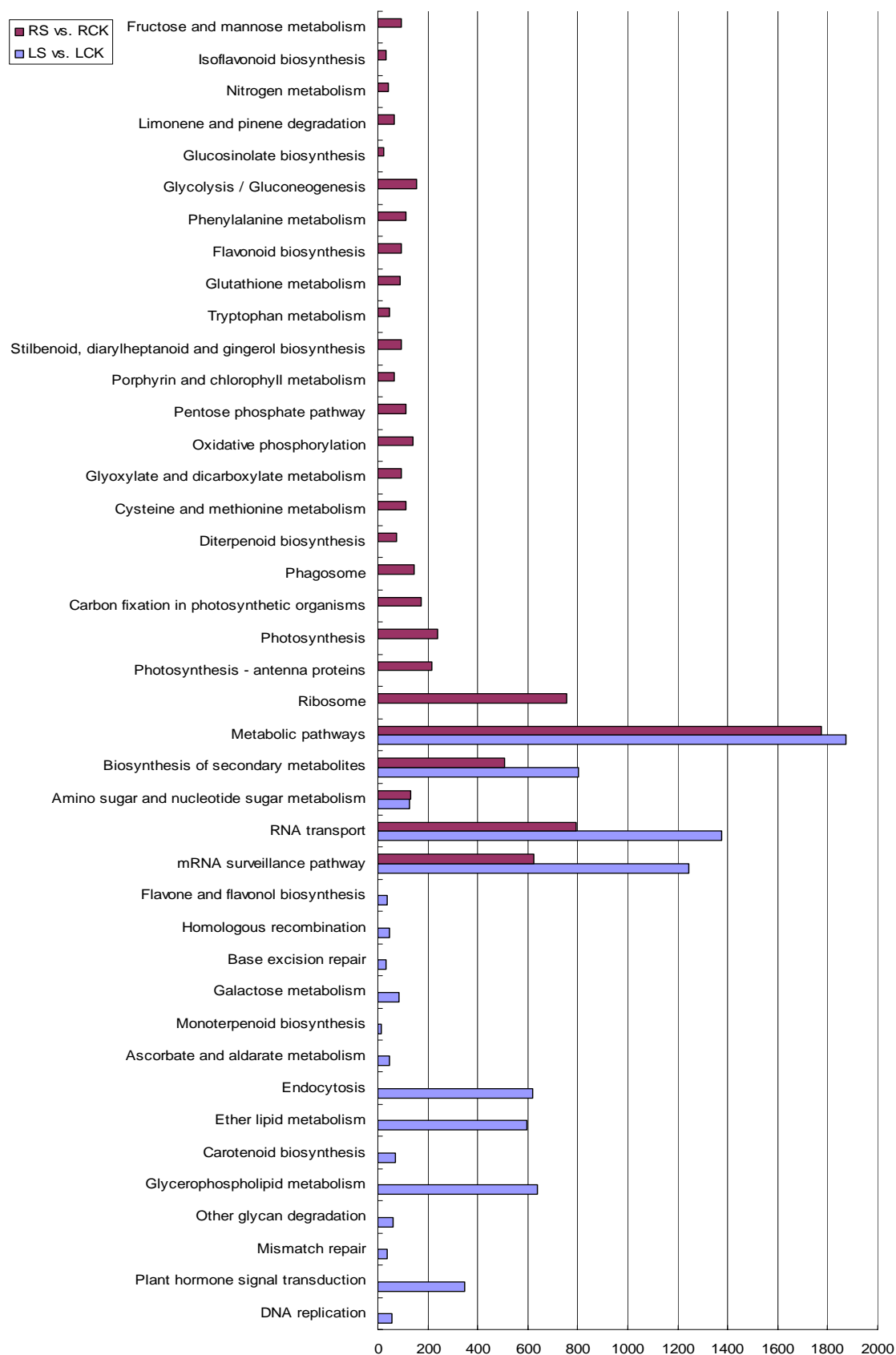

**S3 Fig. The subcategories of over-represented KO terms ( $Q$ -value < 0.05) in the LS vs. LCK and RS vs. RCK.**

Supplement: S3 Fig — (PDF) [file pone.0146242.s003.pdf]
