# Supplementary material for: Transcriptome Analysis of Salt Stress Responsiveness in the Seedlings of Dongxiang Wild Rice (Oryza rufipogon Griff.)
Source: PLoS One. 2016 Jan 11;11(1):e0146242. doi: 10.1371/journal.pone.0146242 (PMC4709063; doi:10.1371/journal.pone.0146242)
Supplement: S5 Table — (PDF) [file pone.0146242.s008.pdf]

S5 Table. List of CCCH-type ZFP genes among the DEGs detected by RNA-Seq.

Gene name	Gene ID	Up or down (Log_2ratio)	
		LS vs. LCK	RS vs. RCK
OsC3H9	LOC_Os01g45730	none	down (-1.18)
OsC3H10	LOC_Os01g53650	up (12.04)	none
OsC3H20	LOC_Os03g02160	down (-1.47)	none
OsC3H22	LOC_Os03g21140	down (-1.50)	none
OsC3H32	LOC_Os04g57600	up (1.39)	none
OsC3H37	LOC_Os05g45020	none	up (1.23)
OsC3H39	LOC_Os05g50080	down (-1.01)	none
OsC3H42	LOC_Os06g32720	down (-3.78)	none
OsC3H49	LOC_Os07g18050	none	up (1.02)
OsC3H54	LOC_Os08g03310	up (2.49)	none
OsC3H62	LOC_Os10g25220	down (-1.67)	none
