# Supplementary material for: Transcriptome Analysis of Salt Stress Responsiveness in the Seedlings of Dongxiang Wild Rice (Oryza rufipogon Griff.)
Source: PLoS One. 2016 Jan 11;11(1):e0146242. doi: 10.1371/journal.pone.0146242 (PMC4709063; doi:10.1371/journal.pone.0146242)
Supplement: S6 Table — (PDF) [file pone.0146242.s009.pdf]

S6 Table. List of NAC-type genes among the DEGs detected by RNA-Seq.

Gene name	Gene ID	Up or down (Log_2ratio)	
		LS vs. LCK	RS vs. RCK
ONAC001	LOC_Os09g33490	down (-1.12)	none
ONAC003	LOC_Os01g09550	none	down (-1.66)
ONAC007	LOC_Os06g04090	none	down (-1.35)
ONAC008	LOC_Os04g43560	none	down (-1.40)
ONAC010	LOC_Os07g37920	up (1.87)	none
ONAC012	LOC_Os05g37080	none	down (-1.34)
ONAC015	LOC_Os07g48550	down (-1.27)	none
ONAC017	LOC_Os11g05614	none	up (1.36)
ONAC022	LOC_Os03g04070	none	up (1.68)
ONAC029	LOC_Os08g02300	down (-1.12)	none
ONAC039	LOC_Os03g21030	up (1.97)	none
ONAC048	LOC_Os01g66120	up (2.28)	up (1.21)
ONAC055	LOC_Os03g01870	down (-1.93)	none
ONAC062	LOC_Os05g48850	down (-1.11)	down (-1.98)
ONAC066	LOC_Os03g56580	down (-1.52)	up (1.64)
ONAC068	LOC_Os01g60020	none	up (3.35)
