# Supplementary material for: Transcriptome Analysis of Salt Stress Responsiveness in the Seedlings of Dongxiang Wild Rice (Oryza rufipogon Griff.)
Source: PLoS One. 2016 Jan 11;11(1):e0146242. doi: 10.1371/journal.pone.0146242 (PMC4709063; doi:10.1371/journal.pone.0146242)
Supplement: S7 Table — (PDF) [file pone.0146242.s010.pdf]

S7 Table. List of MYB-type TF genes among the DEGs detected by RNA-Seq.

Gene ID	Type	Up or down (Log_2ratio)	
		LS vs. LCK	RS vs. RCK
LOC_Os01g03720	R2R3	up (1.08)	up (1.40)
LOC_Os01g07450	R2R3	none	down (-2.23)
LOC_Os01g16810	R2R3	down (-10.37)	up (1.44)
LOC_Os01g18240	R2R3	none	down (-1.11)
LOC_Os01g19330	R2R3	none	up (1.95)
LOC_Os01g36460	R2R3	down (-1.63)	none
LOC_Os01g49160	R2R3	down (-1.62)	up (1.95)
LOC_Os01g50720	R2R3	down (-2.02)	down (-3.18)
LOC_Os01g51260	R2R3	down (-2.77)	none
LOC_Os01g63160	R2R3	down (-1.66)	none
LOC_Os01g64360	R2R3	up (2.47)	up (1.01)
LOC_Os02g02370	R2R3	none	down (-1.11)
LOC_Os02g36890	R2R3	down (-3.78)	none
LOC_Os02g40530	R2R3	none	up (3.76)
LOC_Os02g42870	R2R3	down (-3.25)	none
LOC_Os03g04900	R2R3	none	down (-1.16)
LOC_Os03g20090	R2R3	up (1.39)	up (1.64)
LOC_Os03g25550	R2R3	down (-1.37)	none
LOC_Os03g26130	R2R3	down (-2.01)	none
LOC_Os03g27090	R2R3	none	down (-1.06)
LOC_Os04g38740	R2R3	down (-2.80)	none
LOC_Os04g45020	R2R3	none	down (-1.48)
LOC_Os05g04210	R2R3	up (9.49)	up (4.60)
LOC_Os05g04820	R2R3	down (-1.16)	down (-1.34)
LOC_Os05g28320	R2R3	down (-2.38)	none
LOC_Os05g35500	R2R3	none	down (-1.07)

LOC_Os06g02250	R2R3	none	down (-2.93)
LOC_Os06g06740	R2R3	down (-9.55)	none
LOC_Os06g10350	R2R3	down (-1.32)	none
LOC_Os06g14670	R2R3	up (1.17)	down (-1.72)
LOC_Os06g43090	R2R3	down (-1.29)	none
LOC_Os07g37210	R2R3	up (1.41)	down (-1.54)
LOC_Os07g44090	R2R3	none	down (-1.51)
LOC_Os07g48870	R2R3	none	up (1.83)
LOC_Os08g33660	R2R3	down (-3.01)	none
LOC_Os08g33800	R2R3	down (-2.67)	none
LOC_Os08g37970	R2R3	down (-1.48)	none
LOC_Os08g43450	R2R3	down (-1.68)	none
LOC_Os09g23620	R2R3	none	up (1.18)
LOC_Os09g26170	R2R3	none	down (-1.34)
LOC_Os09g36250	R2R3	none	down (-1.05)
LOC_Os10g33810	R2R3	up (4.40)	none
LOC_Os12g07640	R2R3	none	down (-2.08)
LOC_Os12g33070	R2R3	down (-2.52)	none
LOC_Os12g37690	R2R3	up (2.63)	up (3.15)
LOC_Os12g37970	R2R3	none	down (-1.41)
LOC_Os12g38400	R2R3	down (-1.44)	none
LOC_Os01g09280	MYB-related	up (1.23)	up (1.48)
LOC_Os01g09640	MYB-related	up (1.33)	none
LOC_Os01g41900	MYB-related	down (-1.05)	up (1.58)
LOC_Os02g46030	MYB-related	up (3.49)	up (1.22)
LOC_Os04g49450	MYB-related	up (4.68)	none
LOC_Os06g51260	MYB-related	up (1.03)	none
LOC_Os08g04840	MYB-related	up (1.37)	none
LOC_Os08g06110	MYB-related	up (1.17)	none

LOC_Os01g43230	MYB-related	up (1.52)	none
LOC_Os06g28630	MYB-related	down (-3.18)	none
LOC_Os01g63460	MYB-related	down (-2.07)	down (-1.71)
LOC_Os01g64360	MYB-related	up (2.47)	up (1.01)
LOC_Os05g37730	MYB-related	none	down (-1.55)
LOC_Os01g12860	R1R2R3	down (-3.32)	none
LOC_Os12g13570	R1R2R3	down (-3.40)	none
