# Supplementary material for: Transcriptome Analysis of Salt Stress Responsiveness in the Seedlings of Dongxiang Wild Rice (Oryza rufipogon Griff.)
Source: PLoS One. 2016 Jan 11;11(1):e0146242. doi: 10.1371/journal.pone.0146242 (PMC4709063; doi:10.1371/journal.pone.0146242)
Supplement: S8 Table — (PDF) [file pone.0146242.s011.pdf]

S8 Table. List of bZIP TF genes among the DEGs detected by RNA-Seq.

Gene name	Gene ID	Group	Up or down (Log_2ratio)	
			LS vs. LCK	RS vs. RCK
OsbZIP02	LOC_Os01g11350	V	down (-3.67)	none
OsbZIP06	LOC_Os01g55150	V	down (-2.78)	none
OsbZIP10	LOC_Os01g64000	VI	none	up (3.20)
OsbZIP11	LOC_Os01g64020	VII	none	up (1.77)
OsbZIP12	LOC_Os01g64730	VI	up (2.51)	up (1.61)
OsbZIP14	LOC_Os02g03960	IV	down (-1.69)	down (-1.01)
OsbZIP16	LOC_Os02g09830	IV	up (1.62)	none
OsbZIP19	LOC_Os02g14910	V	down (-2.49)	down (-1.11)
OsbZIP20	LOC_Os02g16680	IV	up (1.55)	none
OsbZIP24	LOC_Os02g58670	VI	none	up (1.01)
OsbZIP25	LOC_Os03g03550	IX	down (-1.33)	none
OsbZIP32	LOC_Os03g56010	IV	none	up (3.30)
OsbZIP36	LOC_Os04g41820	IX	down (-3.36)	none
OsbZIP37	LOC_Os04g54474	VII	down (-1.45)	none
OsbZIP41	LOC_Os05g37170	VII	none	up (1.53)
OsbZIP45	LOC_Os05g49420	I	up (1.28)	none
OsbZIP58	LOC_Os07g08420	IV	up (3.29)	up (1.32)
OsbZIP61	LOC_Os07g48180	IX	down (-3.19)	none
OsbZIP62	LOC_Os07g48660	VI	down (-1.43)	none
OsbZIP64	LOC_Os08g07970	VII	down (-1.62)	none
OsbZIP67	LOC_Os08g38020	IV	none	up (2.56)
OsbZIP69	LOC_Os08g43600	VI	down (-1.56)	none
OsbZIP73	LOC_Os09g29820	IV	down (-2.39)	none
OsbZIP74	LOC_Os09g31390	VII	none	down (-1.03)
OsbZIP77	LOC_Os09g36910	VI	down (-3.20)	down (-1.59)
OsbZIP79	LOC_Os11g05480	VII	down (-1.51)	none
OsbZIP89	LOC_Os12g43790	IV	none	up (1.56)
