# Supplementary material for: Transcriptome Analysis of Salt Stress Responsiveness in the Seedlings of Dongxiang Wild Rice (Oryza rufipogon Griff.)
Source: PLoS One. 2016 Jan 11;11(1):e0146242. doi: 10.1371/journal.pone.0146242 (PMC4709063; doi:10.1371/journal.pone.0146242)
Supplement: S9 Table — (PDF) [file pone.0146242.s012.pdf]

S9 Table. List of AP2/ERF TF genes among the DEGs detected by RNA-Seq.

Gene ID	Phy. Subfamily	Up or down (Log_2ratio)	
		LS vs. LCK	RS vs. RCK
LOC_Os01g04750	RAV	none	down (-1.35)
LOC_Os01g07120	DREB	up (2.44)	none
LOC_Os01g12440	ERF	down (-1.59)	none
LOC_Os01g46870	ERF	down (-12.37)	none
LOC_Os01g49830	RAV	none	up (1.02)
LOC_Os01g64790	DREB	up (3.31)	up (2.18)
LOC_Os01g67410	AP2	down (-2.81)	none
LOC_Os02g32140	ERF	up (4.21)	up (3.60)
LOC_Os02g40070	AP2	down (-9.87)	none
LOC_Os02g42585	ERF	up (1.86)	none
LOC_Os02g43790	ERF	down (-1.13)	up (1.36)
LOC_Os02g43820	DREB	up (2.67)	none
LOC_Os02g52670	DREB	up (3.33)	up (1.87)
LOC_Os02g55380	ERF	none	up (3.01)
LOC_Os03g08460	ERF	down (-1.10)	none
LOC_Os03g15660	ERF	down (-4.40)	none
LOC_Os03g64260	ERF	none	up (4.68)
LOC_Os04g34970	DREB	up (12.34)	none
LOC_Os04g46250	DREB	none	up (1.33)
LOC_Os04g46400	DREB	down (-1.41)	down (-1.44)
LOC_Os04g55970	AP2	down (-10.10)	none
LOC_Os04g57340	ERF	up (1.14)	none
LOC_Os05g32270	DREB	down (-2.12)	none
LOC_Os05g36100	ERF	none	up (9.65)
LOC_Os05g37640	DREB	up (1.28)	up (1.95)
LOC_Os05g47650	RAV	none	up (1.58)

LOC_Os05g49700	DREB	up (2.04)	none
LOC_Os06g03670	ERF	up (4.64)	none
LOC_Os06g07030	DREB	up (1.89)	up (2.33)
LOC_Os06g08340	ERF	none	up (4.30)
LOC_Os06g40150	ERF	down (-1.78)	none
LOC_Os06g43220	AP2	down (-3.78)	none
LOC_Os06g44750	AP2	down (-10.40)	none
LOC_Os07g03250	ERF	down (-5.81)	none
LOC_Os07g13170	AP2	down (-3.33)	none
LOC_Os08g36920	ERF	none	up (12.22)
LOC_Os08g42550	RAV	down (-2.23)	none
LOC_Os09g39850	ERF	none	up (2.79)
LOC_Os10g41130	DREB	down (-1.03)	none
LOC_Os11g03540	AP2	down (-2.13)	down (-1.37)
LOC_Os12g03290	AP2	down (-1.81)	none
