# Supplementary material for: Transcriptome Analysis of Salt Stress Responsiveness in the Seedlings of Dongxiang Wild Rice (Oryza rufipogon Griff.)
Source: PLoS One. 2016 Jan 11;11(1):e0146242. doi: 10.1371/journal.pone.0146242 (PMC4709063; doi:10.1371/journal.pone.0146242)
Supplement: S10 Table — (PDF) [file pone.0146242.s013.pdf]

**S10 Table. List of TF genes among the significant up-regulated DEGs detected in LS vs. LCK.**

| Gene ID | log2 Ratio(LS/LCK) | Description |
|---|---|---|
| LOC_Os01g56690 | 9.89 | helix-loop-helix DNA-binding domain containing protein |
| LOC_Os08g36740 | 3.16 | bhelix-loop-helix transcription factor |
| LOC_Os02g43330 | 8.44 | Homeodomain-leucine zipper transcription factor |
| LOC_Os04g45810 | 3.97 | Homeodomain-leucine zipper transcription factor |
| LOC_Os09g21180 | 3.29 | homeobox associated leucine zipper |
| LOC_Os01g57890 | 4.85 | Homeobox domain containing protein |
| LOC_Os06g45040 | 4.58 | B-box zinc finger family protein |
| LOC_Os02g49230 | 3.16 | CCT/B-box zinc finger protein |
| LOC_Os07g39960 | 4.52 | ZOS7-07 - C2H2 zinc finger protein |
| LOC_Os03g62230 | 4.32 | ZOS3-24 - C2H2 zinc finger protein |
| LOC_Os01g62190 | 3.67 | ZOS1-15 - C2H2 zinc finger protein |
| LOC_Os03g60570 | 3.65 | ZOS3-22 - C2H2 zinc finger protein |
| LOC_Os02g45780 | 3.28 | zinc finger, C3HC4 type domain containing protein |
| LOC_Os12g02210 | 3.52 | RING finger protein |
| LOC_Os05g29710 | 3.16 | RING-H2 finger protein |
| LOC_Os02g26430 | 3.78 | WRKY42 |
| LOC_Os06g06360 | 3.45 | WRKY113 |
| LOC_Os01g39020 | 3.74 | HSF-type DNA-binding domain containing protein |
| LOC_Os06g35960 | 3.35 | HSF domain class transcription factor |
| LOC_Os11g04400 | 3.23 | GRAS family transcription factor containing protein |
