# Supplementary material for: Transcriptome Analysis of Salt Stress Responsiveness in the Seedlings of Dongxiang Wild Rice (Oryza rufipogon Griff.)
Source: PLoS One. 2016 Jan 11;11(1):e0146242. doi: 10.1371/journal.pone.0146242 (PMC4709063; doi:10.1371/journal.pone.0146242)
Supplement: S11 Table — (PDF) [file pone.0146242.s014.pdf]

S11 Table. List of TF genes among the significant up-regulated DEGs detected in RS vs. RCK.

Gene ID	log2 Ratio(RS/RCK)	Description
LOC_Os03g32220	11.73	ZOS3-11 - C2H2 zinc finger protein
LOC_Os04g39520	3.93	ZOS4-08 - C2H2 zinc finger protein
LOC_Os11g47700	11.23	zinc finger, C3HC4 type domain containing protein
LOC_Os11g04680	9.88	zinc finger, C3HC4 type domain containing protein
LOC_Os11g47690	4.07	zinc finger, C3HC4 type domain containing protein
LOC_Os03g50310	4.34	CCT/B-box zinc finger protein
LOC_Os02g39710	3.15	CCT/B-box zinc finger protein
LOC_Os06g47850	3.25	zinc finger family protein
LOC_Os12g24870	4.15	SWIM zinc finger family protein
LOC_Os02g33720	3.21	RING-H2 finger protein
LOC_Os01g60490	10.72	WRKY22
LOC_Os05g49620	3.97	WRKY19
LOC_Os01g09100	3.68	WRKY10
LOC_Os02g26430	3.62	WRKY42
LOC_Os12g02400	3.48	WRKY114
LOC_Os06g10820	10.53	helix-loop-helix DNA-binding domain containing protein
LOC_Os01g09930	9.73	BHLH transcription factor
LOC_Os12g32400	3.70	helix-loop-helix DNA-binding domain containing protein
LOC_Os04g23550	3.62	basic helix-loop-helix family protein
LOC_Os02g43330	4.73	homeobox associated leucine zipper
LOC_Os04g45810	3.44	Homeodomain-leucine zipper transcription factor
LOC_Os05g30660	3.18	leucine zipper protein-like
LOC_Os01g21590	3.79	homeodomain
LOC_Os12g10520	10.18	OsMADS33 - MADS-box family gene with MIKCc type-box
LOC_Os01g06560	4.19	transcription factor HBP-1b
LOC_Os11g04400	3.15	GRAS family transcription factor containing protein
