# Supplementary material for: Transcriptome Analysis of Salt Stress Responsiveness in the Seedlings of Dongxiang Wild Rice (Oryza rufipogon Griff.)
Source: PLoS One. 2016 Jan 11;11(1):e0146242. doi: 10.1371/journal.pone.0146242 (PMC4709063; doi:10.1371/journal.pone.0146242)
Supplement: S12 Table — (PDF) [file pone.0146242.s015.pdf]

S12 Table. Significant GO terms of DEGs in the biological process category for LS vs. LCK.

GO term	GO term annotation	*P*-value
GO:0006323	DNA packaging	9.58E-31
GO:0031497	chromatin assembly	4.43E-30
GO:0071103	DNA conformation change	1.88E-29
GO:0006334	nucleosome assembly	2.64E-29
GO:0034728	nucleosome organization	2.64E-29
GO:0065004	protein-DNA complex assembly	1.4E-25
GO:0071824	protein-DNA complex subunit organization	1.4E-25
GO:0006333	chromatin assembly or disassembly	7.53E-23
GO:0007018	microtubule-based movement	1.02E-19
GO:0006928	cellular component movement	8.04E-16
GO:0007017	microtubule-based process	1.19E-15
GO:0022607	cellular component assembly	1E-11
GO:0065003	macromolecular complex assembly	2.22E-11
GO:0034622	cellular macromolecular complex assembly	3.44E-10
GO:0006261	DNA-dependent DNA replication	2.81E-08
GO:0051301	cell division	5.5E-08
GO:0043933	macromolecular complex subunit organization	5.7E-08
GO:0008283	cell proliferation	1.05E-07
GO:0006270	DNA replication initiation	4.05E-07
GO:0006325	chromatin organization	5.17E-07
GO:0007010	cytoskeleton organization	6.29E-06
GO:0000226	microtubule cytoskeleton organization	8.67E-06
GO:0051276	chromosome organization	1.09E-05
GO:0051726	regulation of cell cycle	1.13E-05
GO:0010564	regulation of cell cycle process	0.00003
GO:0006996	organelle organization	3.04E-05
GO:0009415	response to water stimulus	0.000051
GO:0007346	regulation of mitotic cell cycle	9.74E-05
GO:0009865	pollen tube adhesion	9.75E-05
GO:0051258	protein polymerization	0.0001
GO:0051225	spindle assembly	0.00012
GO:0000910	cytokinesis	0.00013
GO:0016043	cellular component organization	0.00016
GO:0009219	pyrimidine deoxyribonucleotide metabolic process	0.00021
GO:0009221	pyrimidine deoxyribonucleotide biosynthetic process	0.00021
GO:0009262	deoxyribonucleotide metabolic process	0.00021
GO:0009263	deoxyribonucleotide biosynthetic process	0.00021
GO:0009265	2'-deoxyribonucleotide biosynthetic process	0.00021
GO:0009394	2'-deoxyribonucleotide metabolic process	0.00021
GO:0019692	deoxyribose phosphate metabolic process	0.00021
GO:0046385	deoxyribose phosphate biosynthetic process	0.00021
GO:1901987	regulation of cell cycle phase transition	0.00041

GO:1901990	regulation of mitotic cell cycle phase transition	0.00041
GO:0007051	spindle organization	0.00055
GO:0022402	cell cycle process	0.00057
GO:0006259	DNA metabolic process	0.00086
GO:0010035	response to inorganic substance	0.00095
GO:0009788	negative regulation of abscisic acid mediated signaling pathway	0.00114
GO:1901420	negative regulation of response to alcohol	0.00114
GO:0007020	microtubule nucleation	0.00121
GO:0009157	deoxyribonucleoside monophosphate biosynthetic process	0.00223
GO:0009162	deoxyribonucleoside monophosphate metabolic process	0.00223
GO:0009176	pyrimidine deoxyribonucleoside monophosphate metabolic process	0.00223
GO:0009177	pyrimidine deoxyribonucleoside monophosphate biosynthetic process	0.00223
GO:0048285	organelle fission	0.00276
GO:0006022	aminoglycan metabolic process	0.00395
GO:0009065	glutamine family amino acid catabolic process	0.00395
GO:0000280	nuclear division	0.00555
GO:0033205	cell cycle cytokinesis	0.00575
GO:0000912	assembly of actomyosin apparatus involved in cell cycle cytokinesis	0.0066
GO:0000914	phragmoplast assembly	0.0066
GO:0031032	actomyosin structure organization	0.0066
GO:0016572	histone phosphorylation	0.00684
GO:0010389	regulation of G2/M transition of mitotic cell cycle	0.0101
GO:0050896	response to stimulus	0.01236
GO:0016998	cell wall macromolecule catabolic process	0.01586
GO:0009308	amine metabolic process	0.01709
GO:0000087	M phase of mitotic cell cycle	0.0203
GO:0007067	mitosis	0.0203
GO:0000911	cytokinesis by cell plate formation	0.02187
GO:0010466	negative regulation of peptidase activity	0.02232
GO:0051346	negative regulation of hydrolase activity	0.02232
GO:0052547	regulation of peptidase activity	0.02232
GO:0051567	histone H3-K9 methylation	0.03359
GO:0006026	aminoglycan catabolic process	0.03747
GO:0006030	chitin metabolic process	0.03747
GO:0006032	chitin catabolic process	0.03747
GO:0046348	amino sugar catabolic process	0.03747
GO:1901072	glucosamine-containing compound catabolic process	0.03747
GO:0048443	stamen development	0.0417
GO:0048466	androecium development	0.0417
GO:0051273	beta-glucan metabolic process	0.04946
