# Supplementary material for: Transcriptome Analysis of Salt Stress Responsiveness in the Seedlings of Dongxiang Wild Rice (Oryza rufipogon Griff.)
Source: PLoS One. 2016 Jan 11;11(1):e0146242. doi: 10.1371/journal.pone.0146242 (PMC4709063; doi:10.1371/journal.pone.0146242)
Supplement: S13 Table — (PDF) [file pone.0146242.s016.pdf]

S13 Table. Significant GO terms of DEGs in the biological process category for RS vs. RCK.

GO term	GO term annotation	*P*-value
GO:0015979	photosynthesis	1.5E-204
GO:0006412	translation	3.9E-198
GO:0019684	photosynthesis, light reaction	2.6E-110
GO:0009765	photosynthesis, light harvesting	8.1E-109
GO:1901576	organic substance biosynthetic process	5.72E-92
GO:0006091	generation of precursor metabolites and energy	1.88E-90
GO:0009058	biosynthetic process	1.4E-87
GO:0010467	gene expression	4.94E-84
GO:0044249	cellular biosynthetic process	1.37E-83
GO:0034645	cellular macromolecule biosynthetic process	7.1E-81
GO:0009059	macromolecule biosynthetic process	4.79E-80
GO:0010218	response to far red light	1.39E-42
GO:0042254	ribosome biogenesis	4.69E-42
GO:0010114	response to red light	5.41E-42
GO:0022613	ribonucleoprotein complex biogenesis	6.81E-41
GO:0010207	photosystem II assembly	5.42E-34
GO:0010038	response to metal ion	9.07E-33
GO:0006414	translational elongation	3.21E-32
GO:0019685	photosynthesis, dark reaction	5.65E-31
GO:0015977	carbon fixation	7.39E-30
GO:0019253	reductive pentose-phosphate cycle	2.57E-29
GO:0046686	response to cadmium ion	7.13E-28
GO:0009628	response to abiotic stimulus	1.35E-27
GO:0009637	response to blue light	1.75E-27
GO:0008152	metabolic process	2.38E-27
GO:0044237	cellular metabolic process	8.64E-27
GO:0006364	rRNA processing	1.16E-26
GO:0044085	cellular component biogenesis	1.91E-25
GO:0016072	rRNA metabolic process	2.25E-25
GO:0009409	response to cold	1.2E-24
GO:0009266	response to temperature stimulus	9.33E-23
GO:0043623	cellular protein complex assembly	7.13E-22
GO:0034622	cellular macromolecular complex assembly	1.73E-21
GO:0010035	response to inorganic substance	5.46E-21
GO:0009639	response to red or far red light	3.75E-20
GO:0015995	chlorophyll biosynthetic process	2.93E-19
GO:0055114	oxidation-reduction process	3.13E-19
GO:0006818	hydrogen transport	6.88E-19
GO:0035304	regulation of protein dephosphorylation	9.32E-19
GO:0015992	proton transport	1.15E-18
GO:0065003	macromolecular complex assembly	2.43E-18
GO:0006461	protein complex assembly	4.61E-18

GO:0070271	protein complex biogenesis	5.79E-18
GO:0019344	cysteine biosynthetic process	1.65E-17
GO:0035303	regulation of dephosphorylation	3.03E-17
GO:0044710	single-organism metabolic process	5.1E-17
GO:0044272	sulfur compound biosynthetic process	5.89E-17
GO:0006534	cysteine metabolic process	1.1E-16
GO:0019318	hexose metabolic process	2.28E-16
GO:0009070	serine family amino acid biosynthetic process	2.37E-16
GO:0009416	response to light stimulus	6.08E-16
GO:0006006	glucose metabolic process	6.2E-15
GO:0042274	ribosomal small subunit biogenesis	7.06E-15
GO:0009767	photosynthetic electron transport chain	8.64E-15
GO:0009773	photosynthetic electron transport in photosystem I	2.96E-14
GO:0000097	sulfur amino acid biosynthetic process	3.11E-14
GO:0015994	chlorophyll metabolic process	4.97E-14
GO:0000096	sulfur amino acid metabolic process	9.57E-14
GO:0006779	porphyrin-containing compound biosynthetic process	2.04E-13
GO:0022607	cellular component assembly	3.29E-13
GO:0044267	cellular protein metabolic process	3.65E-13
GO:1901659	glycosyl compound biosynthetic process	5.07E-13
GO:0005996	monosaccharide metabolic process	5.49E-13
GO:0019320	hexose catabolic process	5.92E-13
GO:0046365	monosaccharide catabolic process	5.92E-13
GO:0009069	serine family amino acid metabolic process	6.78E-13
GO:0033014	tetrapyrrole biosynthetic process	7.12E-13
GO:0034470	ncRNA processing	1.07E-12
GO:0006007	glucose catabolic process	1.36E-12
GO:0009314	response to radiation	1.83E-12
GO:0016052	carbohydrate catabolic process	3.88E-12
GO:0080167	response to karrikin	4.39E-12
GO:0010196	nonphotochemical quenching	7.88E-12
GO:1990066	energy quenching	7.88E-12
GO:0005975	carbohydrate metabolic process	2.45E-11
GO:0022900	electron transport chain	2.65E-11
GO:0043933	macromolecular complex subunit organization	3.27E-11
GO:0015985	energy coupled proton transport, down electrochemical gradient	4E-11
GO:0015986	ATP synthesis coupled proton transport	4E-11
GO:0009651	response to salt stress	8.66E-11
GO:0006790	sulfur compound metabolic process	1.37E-10
GO:0006950	response to stress	1.91E-10
GO:0046148	pigment biosynthetic process	1.99E-10
GO:0010466	negative regulation of peptidase activity	2.4E-10
GO:0051346	negative regulation of hydrolase activity	2.4E-10
GO:0052547	regulation of peptidase activity	2.4E-10

GO:0071822	protein complex subunit organization	4.18E-10
GO:1901566	organonitrogen compound biosynthetic process	5.81E-10
GO:0009744	response to sucrose stimulus	7.98E-10
GO:0034285	response to disaccharide stimulus	7.98E-10
GO:0006778	porphyrin-containing compound metabolic process	8.2E-10
GO:0046039	GTP metabolic process	1.02E-09
GO:0001510	RNA methylation	1.27E-09
GO:0033013	tetrapyrrole metabolic process	1.32E-09
GO:0006739	NADP metabolic process	2.75E-09
GO:0006970	response to osmotic stress	4.11E-09
GO:0006740	NADPH regeneration	4.87E-09
GO:0042451	purine nucleoside biosynthetic process	6.84E-09
GO:0046129	purine ribonucleoside biosynthetic process	6.84E-09
GO:0044724	single-organism carbohydrate catabolic process	8.21E-09
GO:0019682	glyceraldehyde-3-phosphate metabolic process	1.33E-08
GO:0019288	isopentenyl diphosphate biosynthetic process, mevalonate-independent pathway	1.55E-08
GO:0006184	GTP catabolic process	1.78E-08
GO:1901069	guanosine-containing compound catabolic process	1.78E-08
GO:0042440	pigment metabolic process	1.98E-08
GO:0009240	isopentenyl diphosphate biosynthetic process	2.3E-08
GO:0046490	isopentenyl diphosphate metabolic process	2.3E-08
GO:0006720	isoprenoid metabolic process	2.56E-08
GO:0006090	pyruvate metabolic process	3.07E-08
GO:0006098	pentose-phosphate shunt	3.68E-08
GO:0009657	plastid organization	6.4E-08
GO:1901605	alpha-amino acid metabolic process	8.58E-08
GO:0050896	response to stimulus	8.94E-08
GO:1901564	organonitrogen compound metabolic process	1.18E-07
GO:0009617	response to bacterium	1.18E-07
GO:0009163	nucleoside biosynthetic process	1.49E-07
GO:0042455	ribonucleoside biosynthetic process	1.49E-07
GO:0051501	diterpene phytoalexin metabolic process	1.54E-07
GO:0009145	purine nucleoside triphosphate biosynthetic process	2.02E-07
GO:0009206	purine ribonucleoside triphosphate biosynthetic process	2.02E-07
GO:0009768	photosynthesis, light harvesting in photosystem I	2.09E-07
GO:0008299	isoprenoid biosynthetic process	4.08E-07
GO:0006733	oxidoreduction coenzyme metabolic process	4.43E-07
GO:1901068	guanosine-containing compound metabolic process	6.04E-07
GO:0015672	monovalent inorganic cation transport	7.1E-07
GO:0010310	regulation of hydrogen peroxide metabolic process	7.52E-07
GO:0046496	nicotinamide nucleotide metabolic process	8.47E-07
GO:0019362	pyridine nucleotide metabolic process	1.04E-06
GO:0034599	cellular response to oxidative stress	1.06E-06

GO:2000377	regulation of reactive oxygen species metabolic process	1.18E-06
GO:0006979	response to oxidative stress	1.78E-06
GO:0009201	ribonucleoside triphosphate biosynthetic process	1.79E-06
GO:0034614	cellular response to reactive oxygen species	1.92E-06
GO:0072524	pyridine-containing compound metabolic process	2.28E-06
GO:0042549	photosystem II stabilization	2.85E-06
GO:0016101	diterpenoid metabolic process	3.43E-06
GO:0019438	aromatic compound biosynthetic process	3.51E-06
GO:0052314	phytoalexin metabolic process	3.71E-06
GO:0006754	ATP biosynthetic process	5.21E-06
GO:0051186	cofactor metabolic process	5.26E-06
GO:0009142	nucleoside triphosphate biosynthetic process	5.48E-06
GO:0044281	small molecule metabolic process	8.52E-06
GO:0006096	glycolysis	8.75E-06
GO:1901607	alpha-amino acid biosynthetic process	1.06E-05
GO:0044550	secondary metabolite biosynthetic process	1.22E-05
GO:0007030	Golgi organization	1.48E-05
GO:0030490	maturation of SSU-rRNA	2.05E-05
GO:0055086	nucleobase-containing small molecule metabolic process	2.13E-05
GO:0000462	maturation of SSU-rRNA from tricistronic rRNA transcript (SSU-rRNA, 5.8S rRNA, LSU-rRNA)	2.38E-05
GO:0044723	single-organism carbohydrate metabolic process	3.01E-05
GO:0072522	purine-containing compound biosynthetic process	3.17E-05
GO:0042548	regulation of photosynthesis, light reaction	0.000037
GO:0016051	carbohydrate biosynthetic process	4.67E-05
GO:0006833	water transport	5.43E-05
GO:0042044	fluid transport	5.43E-05
GO:0042742	defense response to bacterium	5.74E-05
GO:0010205	photoinhibition	8.07E-05
GO:0043155	negative regulation of photosynthesis, light reaction	8.07E-05
GO:0009117	nucleotide metabolic process	8.21E-05
GO:0008652	cellular amino acid biosynthetic process	8.45E-05
GO:0071493	cellular response to UV-B	0.000092
GO:0043467	regulation of generation of precursor metabolites and energy	9.35E-05
GO:0034660	ncRNA metabolic process	0.00011
GO:0009814	defense response, incompatible interaction	0.00012
GO:0009668	plastid membrane organization	0.00012
GO:0010027	thylakoid membrane organization	0.00012
GO:0006753	nucleoside phosphate metabolic process	0.00014
GO:0042221	response to chemical stimulus	0.00015
GO:0009902	chloroplast relocation	0.00017
GO:0051667	establishment of plastid localization	0.00017
GO:0042744	hydrogen peroxide catabolic process	0.00018
GO:0006081	cellular aldehyde metabolic process	0.00019

GO:0009817	defense response to fungus, incompatible interaction	0.00019
GO:0051644	plastid localization	0.0002
GO:0019538	protein metabolic process	0.0002
GO:0010155	regulation of proton transport	0.00021
GO:0016053	organic acid biosynthetic process	0.00022
GO:0046394	carboxylic acid biosynthetic process	0.00022
GO:0009743	response to carbohydrate stimulus	0.00031
GO:0034644	cellular response to UV	0.00036
GO:0070301	cellular response to hydrogen peroxide	0.00047
GO:0019752	carboxylic acid metabolic process	0.00049
GO:0016144	S-glycoside biosynthetic process	0.00057
GO:0019758	glycosinolate biosynthetic process	0.00057
GO:0019761	glucosinolate biosynthetic process	0.00057
GO:0051188	cofactor biosynthetic process	0.00085
GO:0046677	response to antibiotic	0.0009
GO:0051656	establishment of organelle localization	0.00105
GO:0006520	cellular amino acid metabolic process	0.00122
GO:0051502	diterpene phytoalexin biosynthetic process	0.00146
GO:0071669	plant-type cell wall organization or biogenesis	0.00158
GO:0019748	secondary metabolic process	0.00164
GO:1901657	glycosyl compound metabolic process	0.00191
GO:0006026	aminoglycan catabolic process	0.00194
GO:0006030	chitin metabolic process	0.00194
GO:0006032	chitin catabolic process	0.00194
GO:0046348	amino sugar catabolic process	0.00194
GO:1901072	glucosamine-containing compound catabolic process	0.00194
GO:0006721	terpenoid metabolic process	0.00195
GO:0009644	response to high light intensity	0.00203
GO:0044711	single-organism biosynthetic process	0.00207
GO:0009987	cellular process	0.00239
GO:0016102	diterpenoid biosynthetic process	0.00278
GO:0071248	cellular response to metal ion	0.00285
GO:0043436	oxoacid metabolic process	0.00292
GO:0006082	organic acid metabolic process	0.00318
GO:0050832	defense response to fungus	0.00326
GO:0009642	response to light intensity	0.00331
GO:0046688	response to copper ion	0.00333
GO:0009871	jasmonic acid and ethylene-dependent systemic resistance, ethylene mediated signaling pathway	0.00342
GO:0006556	S-adenosylmethionine biosynthetic process	0.00348
GO:0042537	benzene-containing compound metabolic process	0.00582
GO:0006022	aminoglycan metabolic process	0.00591
GO:1901071	glucosamine-containing compound metabolic process	0.00591
GO:0009664	plant-type cell wall organization	0.00653

GO:1901362	organic cyclic compound biosynthetic process	0.00716
GO:0009152	purine ribonucleotide biosynthetic process	0.0097
GO:0018130	heterocycle biosynthetic process	0.01039
GO:0044271	cellular nitrogen compound biosynthetic process	0.01132
GO:0009611	response to wounding	0.01204
GO:0009595	detection of biotic stimulus	0.01236
GO:0009697	salicylic acid biosynthetic process	0.01422
GO:0009696	salicylic acid metabolic process	0.01479
GO:0015988	energy coupled proton transmembrane transport, against electrochemical gradient	0.01572
GO:0015991	ATP hydrolysis coupled proton transport	0.01572
GO:0006164	purine nucleotide biosynthetic process	0.01613
GO:0005992	trehalose biosynthetic process	0.01673
GO:0046351	disaccharide biosynthetic process	0.01704
GO:0009862	systemic acquired resistance, salicylic acid mediated signaling pathway	0.02249
GO:0000028	ribosomal small subunit assembly	0.02784
GO:0010270	photosystem II oxygen evolving complex assembly	0.0312
GO:0016114	terpenoid biosynthetic process	0.03471
GO:0042255	ribosome assembly	0.0398
GO:0071241	cellular response to inorganic substance	0.0398
GO:0046500	S-adenosylmethionine metabolic process	0.04662
GO:0071281	cellular response to iron ion	0.04879
GO:0071704	organic substance metabolic process	0.04929
