# Supplementary material for: Transcriptome Analysis of Salt Stress Responsiveness in the Seedlings of Dongxiang Wild Rice (Oryza rufipogon Griff.)
Source: PLoS One. 2016 Jan 11;11(1):e0146242. doi: 10.1371/journal.pone.0146242 (PMC4709063; doi:10.1371/journal.pone.0146242)
Supplement: S14 Table — (PDF) [file pone.0146242.s017.pdf]

S14 Table. Significant GO terms of DEGs in the cellular component category for LS vs. LCK.

GO term	GO term annotation	*P*-value
GO:0032993	protein-DNA complex	1.76E-34
GO:0016023	cytoplasmic membrane-bounded vesicle	3.78E-31
GO:0031410	cytoplasmic vesicle	3.96E-31
GO:0031988	membrane-bounded vesicle	4.16E-31
GO:0031982	vesicle	4.17E-31
GO:0000786	nucleosome	2.11E-29
GO:0044427	chromosomal part	1.05E-26
GO:0000785	chromatin	1.60E-25
GO:0005694	chromosome	3.49E-18
GO:0009505	plant-type cell wall	3.74E-13
GO:0005874	microtubule	5.25E-13
GO:0005576	extracellular region	1.79E-12
GO:0046658	anchored to plasma membrane	9.17E-12
GO:0015630	microtubule cytoskeleton	2.84E-11
GO:0031226	intrinsic to plasma membrane	3.99E-11
GO:0044430	cytoskeletal part	1.05E-10
GO:0005856	cytoskeleton	3.59E-10
GO:0044459	plasma membrane part	9.89E-08
GO:0005618	cell wall	3.81E-07
GO:0030312	external encapsulating structure	5.48E-06
GO:0031225	anchored to membrane	1.85E-05
GO:0030894	replisome	6.83E-05
GO:0042555	MCM complex	0.00051
GO:0043601	nuclear replisome	0.00051
GO:0005875	microtubule associated complex	0.00087
GO:0009504	cell plate	0.00168
GO:0043596	nuclear replication fork	0.00702
GO:0005657	replication fork	0.00763
GO:0044454	nuclear chromosome part	0.01119
GO:0071944	cell periphery	0.01229
