# Supplementary material for: Transcriptome Analysis of Salt Stress Responsiveness in the Seedlings of Dongxiang Wild Rice (Oryza rufipogon Griff.)
Source: PLoS One. 2016 Jan 11;11(1):e0146242. doi: 10.1371/journal.pone.0146242 (PMC4709063; doi:10.1371/journal.pone.0146242)
Supplement: S15 Table — (PDF) [file pone.0146242.s018.pdf]

S15 Table. Significant GO terms of DEGs in the cellular component category for RS vs. RCK.

GO term	GO term annotation	*P*-value
GO:0005840	ribosome	6.37E-258
GO:0030529	ribonucleoprotein complex	3.79E-226
GO:0009579	thylakoid	3.37E-217
GO:0022626	cytosolic ribosome	8.70E-203
GO:0044445	cytosolic part	1.80E-192
GO:0043228	non-membrane-bounded organelle	1.49E-191
GO:0043232	intracellular non-membrane-bounded organelle	1.49E-191
GO:0044436	thylakoid part	3.27E-183
GO:0034357	photosynthetic membrane	1.70E-180
GO:0044391	ribosomal subunit	3.82E-165
GO:0042651	thylakoid membrane	7.39E-147
GO:0032991	macromolecular complex	5.81E-146
GO:0009507	chloroplast	1.06E-145
GO:0031984	organelle subcompartment	3.93E-141
GO:0009534	chloroplast thylakoid	4.70E-141
GO:0031976	plastid thylakoid	4.70E-141
GO:0009535	chloroplast thylakoid membrane	1.47E-140
GO:0055035	plastid thylakoid membrane	3.30E-140
GO:0044446	intracellular organelle part	1.09E-135
GO:0044422	organelle part	2.32E-135
GO:0009521	photosystem	8.13E-106
GO:0044435	plastid part	9.41E-102
GO:0044434	chloroplast part	2.17E-101
GO:0005829	cytosol	9.61E-96
GO:0005730	nucleolus	2.09E-94
GO:0022625	cytosolic large ribosomal subunit	5.01E-88
GO:0015934	large ribosomal subunit	1.18E-86
GO:0044444	cytoplasmic part	6.34E-80
GO:0005737	cytoplasm	8.05E-77
GO:0010287	plastoglobule	2.50E-75
GO:0015935	small ribosomal subunit	1.95E-74
GO:0009526	plastid envelope	1.32E-71
GO:0009941	chloroplast envelope	5.28E-71
GO:0009522	photosystem I	3.01E-67
GO:0009532	plastid stroma	3.39E-67
GO:0009570	chloroplast stroma	3.43E-65
GO:0009536	plastid	1.84E-62
GO:0022627	cytosolic small ribosomal subunit	2.73E-60
GO:0005618	cell wall	3.59E-56
GO:0048046	apoplast	6.66E-55
GO:0030312	external encapsulating structure	1.55E-52
GO:0005576	extracellular region	1.09E-51

GO:0031967	organelle envelope	3.61E-51
GO:0031981	nuclear lumen	4.83E-51
GO:0009523	photosystem II	2.17E-50
GO:0070013	intracellular organelle lumen	3.01E-50
GO:0043233	organelle lumen	3.38E-50
GO:0031974	membrane-enclosed lumen	3.78E-49
GO:0031975	envelope	1.94E-48
GO:0044428	nuclear part	1.45E-40
GO:0016020	membrane	9.91E-38
GO:0009538	photosystem I reaction center	2.21E-29
GO:0043229	intracellular organelle	6.4E-28
GO:0043226	organelle	7.29E-28
GO:0010319	stromule	1.48E-27
GO:0031977	thylakoid lumen	7.66E-27
GO:0005773	vacuole	8.34E-23
GO:0044424	intracellular part	2.08E-21
GO:0009654	oxygen evolving complex	6.7E-21
GO:0009503	thylakoid light-harvesting complex	1.6E-18
GO:0030076	light-harvesting complex	4.1E-18
GO:0071944	cell periphery	6.09E-17
GO:0005622	intracellular	8.57E-16
GO:0009783	photosystem II antenna complex	7.04E-15
GO:0045261	proton-transporting ATP synthase complex, catalytic core F(1)	1.11E-13
GO:0033178	proton-transporting two-sector ATPase complex, catalytic domain	1.8E-13
GO:0031982	vesicle	2.35E-13
GO:0031988	membrane-bounded vesicle	2.98E-13
GO:0045259	proton-transporting ATP synthase complex	5.64E-13
GO:0016023	cytoplasmic membrane-bounded vesicle	7.56E-13
GO:0031410	cytoplasmic vesicle	7.8E-13
GO:0009543	chloroplast thylakoid lumen	9.69E-13
GO:0031978	plastid thylakoid lumen	9.69E-13
GO:0009517	PSII associated light-harvesting complex II	2.18E-11
GO:0016469	proton-transporting two-sector ATPase complex	5.06E-11
GO:0009533	chloroplast stromal thylakoid	6.75E-11
GO:0005623	cell	2.3E-08
GO:0044464	cell part	2.3E-08
GO:0005886	plasma membrane	2.87E-08
GO:0000275	mitochondrial proton-transporting ATP synthase complex, catalytic core F(1)	0.00000405
GO:0030686	90S preribosome	0.0000113
GO:0005754	mitochondrial proton-transporting ATP synthase, catalytic core	0.0000165
GO:0045267	proton-transporting ATP synthase, catalytic core	0.0000165
GO:0009512	cytochrome b6f complex	0.0000458
GO:0005753	mitochondrial proton-transporting ATP synthase complex	0.0000954
GO:0030684	preribosome	0.00103

GO:0009573	chloroplast ribulose bisphosphate carboxylase complex	0.0011
GO:0048492	ribulose bisphosphate carboxylase complex	0.0011
GO:0043231	intracellular membrane-bounded organelle	0.00126
GO:0031012	extracellular matrix	0.00129
GO:0043227	membrane-bounded organelle	0.00133
GO:0009782	photosystem I antenna complex	0.00237
GO:0005856	cytoskeleton	0.0024
GO:0030054	cell junction	0.00369
GO:0005911	cell-cell junction	0.00458
GO:0009506	plasmodesma	0.00458
GO:0055044	symplast	0.00458
GO:0044437	vacuolar part	0.02749
GO:0030095	chloroplast photosystem II	0.02822
GO:0032040	small-subunit processome	0.03088
GO:0005884	actin filament	0.03291
