# Supplementary material for: Transcriptome Analysis of Salt Stress Responsiveness in the Seedlings of Dongxiang Wild Rice (Oryza rufipogon Griff.)
Source: PLoS One. 2016 Jan 11;11(1):e0146242. doi: 10.1371/journal.pone.0146242 (PMC4709063; doi:10.1371/journal.pone.0146242)
Supplement: S16 Table — (PDF) [file pone.0146242.s019.pdf]

S16 Table. Significant GO terms of DEGs in molecular function category for LS vs. LCK.

GO term	GO term annotation	*P*-value
GO:0046982	protein heterodimerization activity	2.36E-18
GO:0016798	hydrolase activity, acting on glycosyl bonds	1.33E-13
GO:0004553	hydrolase activity, hydrolyzing O-glycosyl compounds	3.21E-13
GO:0003677	DNA binding	8.58E-12
GO:0046983	protein dimerization activity	2.17E-10
GO:0005200	structural constituent of cytoskeleton	3.61E-10
GO:0004857	enzyme inhibitor activity	1.58E-08
GO:0042973	glucan endo-1,3-beta-D-glucosidase activity	2.43E-07
GO:0003777	microtubule motor activity	1.32E-06
GO:0003774	motor activity	2.09E-06
GO:0052692	raffinose alpha-galactosidase activity	5.77E-06
GO:0004867	serine-type endopeptidase inhibitor activity	8.31E-06
GO:0016762	xyloglucan:xyloglucosyl transferase activity	2.02E-05
GO:0030414	peptidase inhibitor activity	3.22E-05
GO:0061134	peptidase regulator activity	3.22E-05
GO:0016760	cellulose synthase (UDP-forming) activity	4.08E-05
GO:0004866	endopeptidase inhibitor activity	8.24E-05
GO:0061135	endopeptidase regulator activity	8.24E-05
GO:0016491	oxidoreductase activity	0.00024
GO:0016757	transferase activity, transferring glycosyl groups	0.00072
GO:0016759	cellulose synthase activity	0.00091
GO:0005515	protein binding	0.00107
GO:0004568	chitinase activity	0.00232
GO:0005506	iron ion binding	0.00304
GO:0003979	UDP-glucose 6-dehydrogenase activity	0.00757
GO:0008061	chitin binding	0.01413
GO:0097367	carbohydrate derivative binding	0.01413
GO:0030234	enzyme regulator activity	0.01458
GO:0016231	beta-N-acetylglucosaminidase activity	0.01667
GO:0034387	4-aminobutyrate:pyruvate transaminase activity	0.01789
GO:0050378	UDP-glucuronate 4-epimerase activity	0.01789
GO:0009055	electron carrier activity	0.02568
GO:0016645	oxidoreductase activity, acting on the CH-NH group of donors	0.03511
GO:0008422	beta-glucosidase activity	0.04118
GO:0004014	adenosylmethionine decarboxylase activity	0.0444
