# Supplementary material for: Transcriptome Analysis of Salt Stress Responsiveness in the Seedlings of Dongxiang Wild Rice (Oryza rufipogon Griff.)
Source: PLoS One. 2016 Jan 11;11(1):e0146242. doi: 10.1371/journal.pone.0146242 (PMC4709063; doi:10.1371/journal.pone.0146242)
Supplement: S17 Table — (PDF) [file pone.0146242.s020.pdf]

S17 Table. Significant GO terms of DEGs in molecular function category for RS vs. RCK.

GO term	GO term annotation	*P*-value
GO:0005198	structural molecule activity	2.2E-249
GO:0003735	structural constituent of ribosome	8.4E-240
GO:0005507	copper ion binding	4.77E-32
GO:0019843	rRNA binding	4.52E-30
GO:0016168	chlorophyll binding	1.09E-20
GO:0016491	oxidoreductase activity	4.79E-18
GO:0008187	poly-pyrimidine tract binding	2.4E-17
GO:0008266	poly(U) RNA binding	2.4E-17
GO:0003746	translation elongation factor activity	4.97E-17
GO:0030414	peptidase inhibitor activity	5.37E-15
GO:0061134	peptidase regulator activity	5.37E-15
GO:0046906	tetrapyrrole binding	1.06E-14
GO:0004866	endopeptidase inhibitor activity	1.39E-14
GO:0061135	endopeptidase regulator activity	1.39E-14
GO:0004867	serine-type endopeptidase inhibitor activity	3.47E-14
GO:0016984	ribulose-bisphosphate carboxylase activity	3.94E-14
GO:0044769	ATPase activity, coupled to transmembrane movement of ions, rotational mechanism	6.97E-13
GO:0003727	single-stranded RNA binding	9.6E-13
GO:0005200	structural constituent of cytoskeleton	2.59E-11
GO:0003723	RNA binding	8.79E-11
GO:0046933	proton-transporting ATP synthase activity, rotational mechanism	1.4E-10
GO:0004857	enzyme inhibitor activity	9.46E-09
GO:0016830	carbon-carbon lyase activity	1.54E-08
GO:0003924	GTPase activity	3.19E-08
GO:0015078	hydrogen ion transmembrane transporter activity	5.08E-08
GO:0004497	monooxygenase activity	2.21E-07
GO:0020037	heme binding	3.68E-07
GO:0047100	glyceraldehyde-3-phosphate dehydrogenase (NADP+) (phosphorylating) activity	5.17E-07
GO:0004568	chitinase activity	5.75E-07
GO:0009055	electron carrier activity	1.27E-06
GO:0016851	magnesium chelatase activity	1.17E-05
GO:0051002	ligase activity, forming nitrogen-metal bonds	1.17E-05
GO:0051003	ligase activity, forming nitrogen-metal bonds, forming coordination complexes	1.17E-05
GO:0004332	fructose-bisphosphate aldolase activity	1.32E-05
GO:0034280	ent-sandaracopimaradiene synthase activity	2.08E-05
GO:0008974	phosphoribulokinase activity	2.98E-05
GO:0015077	monovalent inorganic cation transmembrane transporter activity	3.86E-05
GO:0016832	aldehyde-lyase activity	4.41E-05
GO:0008061	chitin binding	5.24E-05
GO:0097367	carbohydrate derivative binding	5.24E-05

GO:0016209	antioxidant activity	7.78E-05
GO:0008135	translation factor activity, nucleic acid binding	0.00016
GO:0008843	endochitinase activity	0.00016
GO:0016829	lyase activity	0.00023
GO:0046961	proton-transporting ATPase activity, rotational mechanism	0.00026
GO:0016831	carboxy-lyase activity	0.00027
GO:0051287	NAD binding	0.00032
GO:0016620	oxidoreductase activity, acting on the aldehyde or oxo group of donors, NAD or NADP as acceptor	0.00041
GO:0004618	phosphoglycerate kinase activity	0.00059
GO:0045158	electron transporter, transferring electrons within cytochrome b6/f complex of photosystem II activity	0.00077
GO:0004478	methionine adenosyltransferase activity	0.00095
GO:0010242	oxygen evolving activity	0.00109
GO:0009496	plastoquinol--plastocyanin reductase activity	0.00138
GO:0052880	oxidoreductase activity, acting on diphenols and related substances as donors, with copper protein as acceptor	0.00138
GO:0016709	oxidoreductase activity, acting on paired donors, with incorporation or reduction of molecular oxygen, NAD(P)H as one donor, and incorporation of one atom of oxygen	0.00152
GO:0034281	ent-isokaurene synthase activity	0.00255
GO:0034282	ent-pimara-8(14),15-diene synthase activity	0.00255
GO:0016705	oxidoreductase activity, acting on paired donors, with incorporation or reduction of molecular oxygen	0.00262
GO:0004601	peroxidase activity	0.00483
GO:0016684	oxidoreductase activity, acting on peroxide as acceptor	0.00483
GO:0019203	carbohydrate phosphatase activity	0.00581
GO:0043169	cation binding	0.0059
GO:0003959	NADPH dehydrogenase activity	0.0062
GO:0004324	ferredoxin-NADP+ reductase activity	0.0062
GO:0046872	metal ion binding	0.00743
GO:0045485	omega-6 fatty acid desaturase activity	0.00912
GO:0016760	cellulose synthase (UDP-forming) activity	0.00972
GO:0042132	fructose 1,6-bisphosphate 1-phosphatase activity	0.01091
GO:0016903	oxidoreductase activity, acting on the aldehyde or oxo group of donors	0.01613
GO:0009678	hydrogen-translocating pyrophosphatase activity	0.01701
GO:0004427	inorganic diphosphatase activity	0.02071
GO:0004013	adenosylhomocysteinase activity	0.02266
GO:0016802	trialkylsulfonium hydrolase activity	0.02266
GO:0016731	oxidoreductase activity, acting on iron-sulfur proteins as donors, NAD or NADP as acceptor	0.0283
GO:0004056	argininosuccinate lyase activity	0.03422
GO:0031409	pigment binding	0.04017
GO:0016231	beta-N-acetylglucosaminidase activity	0.04231
