# Supplementary material for: Transcriptome Analysis of Salt Stress Responsiveness in the Seedlings of Dongxiang Wild Rice (Oryza rufipogon Griff.)
Source: PLoS One. 2016 Jan 11;11(1):e0146242. doi: 10.1371/journal.pone.0146242 (PMC4709063; doi:10.1371/journal.pone.0146242)
Supplement: S18 Table — (PDF) [file pone.0146242.s021.pdf]

S18 Table. The significant GO terms of DEGs for the cellular component category both in the LS vs. LCK and RS vs. RCK.

GO term	GO term annotation
GO:0071944	cell periphery
GO:0005618	cell wall
GO:0016023	cytoplasmic membrane-bounded vesicle
GO:0031410	cytoplasmic vesicle
GO:0005856	cytoskeleton
GO:0030312	external encapsulating structure
GO:0005576	extracellular region
GO:0031988	membrane-bounded vesicle
GO:0031982	vesicle
