# Supplementary material for: Transcriptome Analysis of Salt Stress Responsiveness in the Seedlings of Dongxiang Wild Rice (Oryza rufipogon Griff.)
Source: PLoS One. 2016 Jan 11;11(1):e0146242. doi: 10.1371/journal.pone.0146242 (PMC4709063; doi:10.1371/journal.pone.0146242)
Supplement: S19 Table — (PDF) [file pone.0146242.s022.pdf]

S19 Table. The significant GO terms of DEGs for the molecular function category both in the LS vs. LCK and RS vs. RCK.

GO term	GO term annotation
GO:0004568	chitinase activity
GO:0004857	enzyme inhibitor activity
GO:0004866	endopeptidase inhibitor activity
GO:0004867	serine-type endopeptidase inhibitor activity
GO:0005200	structural constituent of cytoskeleton
GO:0008061	chitin binding
GO:0009055	electron carrier activity
GO:0016231	beta-N-acetylglucosaminidase activity
GO:0016491	oxidoreductase activity
GO:0016760	cellulose synthase (UDP-forming) activity
GO:0030414	peptidase inhibitor activity
GO:0061134	peptidase regulator activity
GO:0061135	endopeptidase regulator activity
GO:0097367	carbohydrate derivative binding
