# Supplementary material for: Transcriptome Analysis of Salt Stress Responsiveness in the Seedlings of Dongxiang Wild Rice (Oryza rufipogon Griff.)
Source: PLoS One. 2016 Jan 11;11(1):e0146242. doi: 10.1371/journal.pone.0146242 (PMC4709063; doi:10.1371/journal.pone.0146242)
Supplement: S20 Table — (PDF) [file pone.0146242.s023.pdf]

S20 Table. Significant KO terms of DEGs in the LS vs. LCK (*Q*-value < 0.05).

KO term	KO annotation	*P*-value	*Q*-value
ko03015	mRNA surveillance pathway	6.595E-58	8.24E-56
ko03013	RNA transport	2.255E-56	1.41E-54
ko03030	DNA replication	3.088E-10	1.29E-08
ko04075	Plant hormone signal transduction	9.608E-09	3E-07
ko00520	Amino sugar and nucleotide sugar metabolism	1.995E-08	4.99E-07
ko03430	Mismatch repair	1.98E-06	4.12E-05
ko00511	Other glycan degradation	4.071E-06	7.27E-05
ko01110	Biosynthesis of secondary metabolites	9.197E-06	0.000144
ko00564	Glycerophospholipid metabolism	2.828E-05	0.000393
ko00906	Carotenoid biosynthesis	3.778E-05	0.000472
ko00565	Ether lipid metabolism	8.546E-05	0.000971
ko04144	Endocytosis	0.0007153	0.007452
ko00053	Ascorbate and aldarate metabolism	0.0008612	0.008281
ko00902	Monoterpenoid biosynthesis	0.0010466	0.009345
ko00052	Galactose metabolism	0.0018897	0.014822
ko01100	Metabolic pathways	0.0018972	0.014822
ko03410	Base excision repair	0.0022255	0.016364
ko03440	Homologous recombination	0.0024781	0.017209
ko00944	Flavone and flavonol biosynthesis	0.0039583	0.026041
