# Supplementary material for: Transcriptome Analysis of Salt Stress Responsiveness in the Seedlings of Dongxiang Wild Rice (Oryza rufipogon Griff.)
Source: PLoS One. 2016 Jan 11;11(1):e0146242. doi: 10.1371/journal.pone.0146242 (PMC4709063; doi:10.1371/journal.pone.0146242)
Supplement: S21 Table — (PDF) [file pone.0146242.s024.pdf]

S21 Table. Significant KO terms of DEGs in the RS vs. RCK (*Q*-value < 0.05).

KO term	KO annotation	*P*-value	*Q*-value
ko03010	Ribosome	1.6E-241	2E-239
ko00196	Photosynthesis - antenna proteins	8.7E-138	5.5E-136
ko00195	Photosynthesis	3E-122	1.3E-120
ko00710	Carbon fixation in photosynthetic organisms	2.75E-35	8.66E-34
ko01110	Biosynthesis of secondary metabolites	6.41E-16	1.62E-14
ko01100	Metabolic pathways	2.26E-11	4.75E-10
ko04145	Phagosome	3.56E-11	6.4E-10
ko00904	Diterpenoid biosynthesis	1.37E-10	2.15E-09
ko00270	Cysteine and methionine metabolism	5.62E-09	7.86E-08
ko00630	Glyoxylate and dicarboxylate metabolism	4.75E-08	5.99E-07
ko00190	Oxidative phosphorylation	1.52E-07	1.66E-06
ko00030	Pentose phosphate pathway	1.58E-07	1.66E-06
ko00860	Porphyrin and chlorophyll metabolism	7.17E-07	6.95E-06
ko03013	RNA transport	6.37E-06	5.73E-05
ko00945	Stilbenoid, diarylheptanoid and gingerol biosynthesis	8.87E-06	7.41E-05
ko00380	Tryptophan metabolism	9.41E-06	7.41E-05
ko00520	Amino sugar and nucleotide sugar metabolism	1.53E-05	0.000113
ko00480	Glutathione metabolism	2.07E-05	0.000145
ko00941	Flavonoid biosynthesis	3.71E-05	0.000246
ko00360	Phenylalanine metabolism	6.99E-05	0.00044
ko00010	Glycolysis / Gluconeogenesis	0.000242	0.001451
ko00966	Glucosinolate biosynthesis	0.000491	0.002814
ko03015	mRNA surveillance pathway	0.000778	0.004263
ko00903	Limonene and pinene degradation	0.001204	0.006322
ko00910	Nitrogen metabolism	0.005067	0.025537
ko00943	Isoflavonoid biosynthesis	0.00671	0.032518
ko00051	Fructose and mannose metabolism	0.007415	0.034604
