# Supplementary material for: Transcriptome Analysis of Salt Stress Responsiveness in the Seedlings of Dongxiang Wild Rice (Oryza rufipogon Griff.)
Source: PLoS One. 2016 Jan 11;11(1):e0146242. doi: 10.1371/journal.pone.0146242 (PMC4709063; doi:10.1371/journal.pone.0146242)
Supplement: S26 Table — (PDF) [file pone.0146242.s029.pdf]

S26 Table . List of the same up-regulated genes among the LS, RS, shoots, and roots.

Gene ID	Description
LOC_Os01g04050	Similar to Wound-induced protease inhibitor (WIP1).
LOC_Os01g04330	Flagellar calcium-binding protein (calflagin) family protein.
LOC_Os01g21250	Late embryogenesis abundant protein 3 family protein.
LOC_Os01g50370	Protein kinase-like domain containing protein.
LOC_Os01g50420	Protein kinase-like domain containing protein.
LOC_Os01g53920	Protein kinase-like domain containing protein.
LOC_Os01g61080	WRKY transcription factor 24 (WRKY24).
LOC_Os01g64360	Putative MYB family transcription factor
LOC_Os01g64470	Harpin-induced 1 domain containing protein.
LOC_Os01g71350	Putative glycosyl hydrolases family 17
LOC_Os02g39330	CHIT1 - Chitinase family protein precursor
LOC_Os02g41840	Protein of unknown function DUF584 family protein.
LOC_Os02g43330	Putative homeobox associated leucine zipper
LOC_Os02g43540	Conserved hypothetical protein.
LOC_Os02g52670	AP2 domain containing protein
LOC_Os03g04110	Putative lysM domain-containing GPI-anchored protein precursor
LOC_Os03g04770	Similar to Beta-amylase PCT-BMYI (EC 3.2.1.2).
LOC_Os03g08320	ZIM domain containing protein
LOC_Os03g11900	Similar to Hexose transporter.
LOC_Os03g12510	Non-symbiotic hemoglobin 2 (rHb2) (ORYsa GLB1b).
LOC_Os03g13300	Similar to Glutamate decarboxylase isozyme 3 (EC 4.1.1.15).
LOC_Os03g15270	Putative gibberellin receptor GID1L2
LOC_Os03g16170	Similar to Protein phosphatase type 2C.
LOC_Os03g18030	Similar to Leucoanthocyanidin dioxygenase-like protein.
LOC_Os03g26910	Similar to Trehalose-6-phosphate phosphatase.
LOC_Os03g32230	ZOS3-12 - C2H2 zinc finger protein
LOC_Os03g50960	LTPL118 - Protease inhibitor/seed storage/LTP family protein precursor
LOC_Os03g57640	Esterase/lipase/thioesterase domain containing protein.
LOC_Os03g60560	ZOS3-21 - C2H2 zinc finger protein
LOC_Os04g45810	Homeodomain-like containing protein.
LOC_Os04g54830	Lambda repressor-like, DNA-binding domain containing protein.
LOC_Os05g29790	Similar to Pectinmethylesterase precursor (EC 3.1.1.11) (Fragment).
LOC_Os05g31670	Putative AWPM-19-like membrane family protein
LOC_Os05g33400	Similar to EDGP.
LOC_Os05g37190	ZOS5-08 - C2H2 zinc finger protein
LOC_Os05g39720	OsWRKY70 - Superfamily of TFs having WRKY and zinc finger domains
LOC_Os06g04230	Conserved hypothetical protein.
LOC_Os06g06360	WRKY DNA-binding domain containing protein
LOC_Os06g25010	Similar to Xylanase inhibitor protein I precursor.
LOC_Os06g44010	OsWRKY28 - Superfamily of TFs having WRKY and zinc finger domains
LOC_Os07g05940	9-cis-epoxycarotenoid dioxygenase 1, chloroplast precursor
LOC_Os07g36560	Transferase family protein.

LOC_Os07g40290	OsGH3.8 - Probable indole-3-acetic acid-amido synthetase
LOC_Os07g48830	Glycosyl transferase 8 domain containing protein
LOC_Os08g28710	Receptor protein kinase CRINKLY4 precursor
LOC_Os09g15670	Similar to Protein phpsphatase 2C (PP2C) (EC 3.1.3.16).
LOC_Os09g17152	OsFBX319 - F-box domain containing protein
LOC_Os09g23590	Conserved hypothetical protein.
LOC_Os10g09240	Conserved hypothetical protein.
LOC_Os10g25290	ZIM domain containing protein
LOC_Os10g25400	GDSL-like lipase/acylhydrolase
LOC_Os10g33990	DUF584 domain containing protein
LOC_Os10g39640	Expansin/Lol pI family protein.
LOC_Os11g06150	Basic proline-rich protein precursor
LOC_Os11g29780	Plant-specific domain TIGR01627 family protein
LOC_Os12g05440	Cytochrome P450 family protein.
LOC_Os12g40260	WD-40 repeat family protein
