# Supplementary material for: Transcriptome Analysis of Salt Stress Responsiveness in the Seedlings of Dongxiang Wild Rice (Oryza rufipogon Griff.)
Source: PLoS One. 2016 Jan 11;11(1):e0146242. doi: 10.1371/journal.pone.0146242 (PMC4709063; doi:10.1371/journal.pone.0146242)
Supplement: S27 Table — (PDF) [file pone.0146242.s030.pdf]

**S27 Table. List of the same down-regulated genes among the LS, RS, shoots, and roots.**

| Gene ID | Description |
|---|---|
| LOC_Os01g47780 | Fasciclin domain containing protein |
| LOC_Os03g02040 | Remorin family protein |
| LOC_Os04g41810 | Cytochrome b561 / ferric reductase transmembrane domain containing protein. |
| LOC_Os04g56060 | Protein kinase domain containing protein |
| LOC_Os05g12580 | Conserved hypothetical protein |
| LOC_Os05g41990 | Peroxidase precursor |
| LOC_Os05g48850 | No apical meristem (NAM) protein |
| LOC_Os11g17720 | Protein of unknown function DUF247, plant family protein. |
