# Supplementary material for: Transcriptome Analysis of Salt Stress Responsiveness in the Seedlings of Dongxiang Wild Rice (Oryza rufipogon Griff.)
Source: PLoS One. 2016 Jan 11;11(1):e0146242. doi: 10.1371/journal.pone.0146242 (PMC4709063; doi:10.1371/journal.pone.0146242)
Supplement: S29 Table — (PDF) [file pone.0146242.s032.pdf]

S29 Table. Primers used for qRT-PCR analysis of differentially expressed genes

Gene ID	Primer	Sequence
LOC_Os03g16320	Os03g16320F	CGCATCATTGGTCGCACTT
	Os03g16320R	GTTCTCCACCGCAAATAGGG
LOC_Os05g12040	Os05g12040F	ATTCATCCGCCACCATTAT
	Os05g12040R	GCCTCTGACCCAACCAAG
LOC_Os02g12380	Os02g12380F	AACAAACAGGGTTATGACAGT
	Os02g12380R	TCGGCTTGAAGATGGAC
LOC_Os09g14860	Os09g14860F	GCAACCCGAACCCAT
	Os09g14860R	CACCACAGCCTTTCCTAA
LOC_Os12g15680	Os12g15680F	AAGGTGAAGACGCTCAAGTA
	Os12g15680R	GTGCGAGGACGAAGAAGT
LOC_Os08g03310	Os08g03310F	CGTGCGTGGACAACATCA
	Os08g03310R	CTGCCAACTGCTTCCTCATC
LOC_Os10g25130	Os10g25130F	AGGAGGAAACCAAATCA
	Os10g25130R	CCTGGCTGTGGCTGTA
LOC_Os01g43120	Os01g43120F	GATTATCCTGATGTCACCCTT
	Os01g43120R	TTGCCCTTCCTTTCCCT
LOC_Os10g36390	Os10g36390F	CTGGACAGTGGCAAGGA
	Os10g36390R	GCTGCGTCGTGTAGGAG
LOC_Os07g05460	Os07g05460F	CGTCGGGCAGCCATTCT
	Os07g05460R	CCCATTGGTGTAGGCGTTTT
LOC_Os01g53750	Os01g53750F	CTATTCAACCGAGTCAGCC
	Os01g53750R	CCGCCAAAGTCACAAGC
LOC_Os12g13570	Os12g13570F	TGGCACAACCATCTTA
	Os12g13570R	GCTCAGCCCATTTATTT
LOC_Os06g12320	Os06g12320F	ATGGGCGTCATCGGCTAC
	Os06g12320R	AGGAGGGCGAACTTGGTG
LOC_Os11g01790	Os11g01790F	ACTGTCGTTAGGCAGGGTG
	Os11g01790R	TTCGCTTGGAAGATTTGGT
LOC_Os04g47080	Os04g47080F	TACAACGGCGAGATAAAGA
	Os04g47080R	CCGAAGGCGTAGGTCA
LOC_Os07g39910	Os07g39910F	GGTTGCTGAAGGCATAGA
	Os07g39910R	CCAGAGCGACCAAGTAAAT
LOC_Os03g55660	Os03g55660F	CCCCTACTGGCGTAACTT
	Os03g55660R	GGGTGATGGAGCAGGACTT
LOC_Os07g12810	Os07g12810F	GCCCAGAGTATGGTCAA
	Os07g12810R	AGTGGTCACGCCCTAAA
LOC_Os10g28970	Os10g28970F	CCACCGCCACCTCCAAA
	Os10g28970R	CATCTTCAGCCGCTCCAC
LOC_Os04g24510	Os04g24510F	GTGCGATGATAACTTGAGC
	Os04g24510R	CATACGGGTTCCCTTGA
LOC_Os06g41670	Os06g41670F	GCAGAAAGGCGGAGAAC
	Os06g41670R	AAACGCTTGGCAATAGA

LOC_Os03g61220	Os03g61220F	CTGTTACTGGCTTTCTGTCTG
	Os03g61220R	CAAATCCCTCGCAATCTC
LOC_Os11g32260	Os11g32260F	ATGATGGTCGTGGTGTT
	Os11g32260R	AAAGCAATAAGAAGAGGG
LOC_Os09g30200	Os09g30200F	TCCATCATTGCTCCACA
	Os09g30200R	CCTCCGCAGATTCATTT
LOC_Os08g03270	Os08g03270F	GCGGCGTCATAGACCACAG
	Os08g03270R	CACCCAGAAGGGCGAAAA
LOC_Os02g19130	Os02g19130F	ATCCGTTCAAGATTGTTAC
	Os02g19130R	CTCCTTCAAGCCCACTA
LOC_Os12g10360	Os12g10360F	CTTGGCAGCATTTGTCTCG
	Os12g10360R	GCAACTCCTCCACATCACC
LOC_Os02g40100	Os02g40100F	ACAGCCCAATGTCCAACTCC
	Os02g40100R	ACACTACCATCTTGTGCTACGC
LOC_Os01g21450	Os01g21450F	AAAACGGTCATTCTCAT
	Os01g21450R	ATCTGGTGCCCTCTTA
LOC_Os05g32110	Os05g32110F	CAAATGCTGCTTCTTCC
	Os05g32110R	GCTTGGGTTGCTCTGC
LOC_Os07g17970	Os07g17970F	ACGAAGAAGCAGGGCAGATA
	Os07g17970R	CAACCGCAGATGATTTACCAA
LOC_Os04g39180	Os04g39180F	AACAGAGGAAAGAACCCAG
	Os04g39180R	GAGGCATCCGAGCAGTA
TUBULIN	TUBULINF	TCAGATGCCCAGTGACAGGA
	TUBULINR	TTGGTGATCTCGGCAACAGA
